# Wide Distribution of Foxicin Biosynthetic Gene Clusters in *Streptomyces* Strains – An Unusual Secondary Metabolite with Various Properties

**DOI:** 10.3389/fmicb.2017.00221

**Published:** 2017-02-21

**Authors:** Anja Greule, Marija Marolt, Denise Deubel, Iris Peintner, Songya Zhang, Claudia Jessen-Trefzer, Christian De Ford, Sabrina Burschel, Shu-Ming Li, Thorsten Friedrich, Irmgard Merfort, Steffen Lüdeke, Philippe Bisel, Michael Müller, Thomas Paululat, Andreas Bechthold

**Affiliations:** ^1^Department of Pharmaceutical Biology and Biotechnology, Albert-Ludwigs-University of FreiburgFreiburg im Breisgau, Germany; ^2^Department of Pharmaceutical and Medical Chemistry, Albert-Ludwigs-University of FreiburgFreiburg im Breisgau, Germany; ^3^Spemann Graduate School of Biology and Medicine, Albert-Ludwigs-University of FreiburgFreiburg im Breisgau, Germany; ^4^Institute of Biochemistry, Albert-Ludwigs-University of FreiburgFreiburg im Breisgau, Germany; ^5^Department of Pharmaceutical Biology, Philipps-University MarburgMarburg, Germany; ^6^Department of Chemistry and Biology, University of SiegenSiegen, Germany

**Keywords:** *Streptomyces*, natural product, foxicin, biosynthetic gene cluster, evolution, siderophore

## Abstract

*Streptomyces diastatochromogenes* Tü6028 is known to produce the polyketide antibiotic polyketomycin. The deletion of the *pokOIV* oxygenase gene led to a non-polyketomycin-producing mutant. Instead, novel compounds were produced by the mutant, which have not been detected before in the wild type strain. Four different compounds were identified and named foxicins A–D. Foxicin A was isolated and its structure was elucidated as an unusual nitrogen-containing quinone derivative using various spectroscopic methods. Through genome mining, the foxicin biosynthetic gene cluster was identified in the draft genome sequence of *S. diastatochromogenes*. The cluster spans 57 kb and encodes three PKS type I modules, one NRPS module and 41 additional enzymes. A *foxBII* gene-inactivated mutant of *S. diastatochromogenes* Tü6028 Δ*pokOIV* is unable to produce foxicins. Homologous *fox* biosynthetic gene clusters were found in more than 20 additional *Streptomyces* strains, overall in about 2.6% of all sequenced *Streptomyces* genomes. However, the production of foxicin-like compounds in these strains has never been described indicating that the clusters are expressed at a very low level or are silent under fermentation conditions. Foxicin A acts as a siderophore through interacting with ferric ions. Furthermore, it is a weak inhibitor of the *Escherichia coli* aerobic respiratory chain and shows moderate antibiotic activity. The wide distribution of the cluster and the various properties of the compound indicate a major role of foxicins in *Streptomyces* strains.

## Introduction

Plants, marine organisms, protozoans, fungi, and bacteria produce a wide range of different secondary metabolites. They are not essential for normal growth, development, or reproduction of an organism, but play a secondary role. There have been several discussions about the selective advantage of these natural products for their producers (Firn and Jones, 2000). Secondary metabolites may act as signals for differentiation, as communication molecules, or as weapons to defend against food competitors (Demain and Fang, 2000) and thus they often possess vital functions in their ecological habitat. The genes responsible for the biosynthesis of a compound are often located next to each other in so called biosynthetic gene clusters. The clusters often span more than 100 kb and encode more than 30 genes related to biosynthesis, transport, regulation, self-resistance and modification. Due to their antibiotic, antitumor, cholesterol-lowering, immunosuppressant or antiviral activities, secondary metabolites are invaluable elements of drug discovery research (Vaishnav and Demain, 2011). Approximately 18.000 bioactive secondary metabolites are produced in bacteria, thereof more than 10.000 compounds are synthesized in *Streptomyces* (Bérdy, 2012). Representatives of the genus *Streptomyces* have been studied extensively in the last decades (Weber et al., 2015b). A well-known class of bioactive secondary metabolites are polyketides that are assembled by modular megaenzymes called PKSs. The subsequent steps in the assembly process are highly similar to the biosynthesis of fatty acids. A detailed introduction to PKS can be found in Staunton and Weissman (2001). Non-ribosomal peptides (NRP) belong to another important class of bioactive compounds. They are synthesized by non-ribosomal peptide synthetase (NRPS) that share certain characteristics with PKS (see Schwarzer et al., 2003 for details).

The genome size of *Streptomyces* ranges from 8 to 9 Mb and shows a high GC (>70%) content. After sequencing the first *Streptomyces* genomes it was noticed that unexpectedly, it contained far more secondary metabolite gene clusters than had been predicted earlier from the numbers of previously identified metabolites (Bentley et al., 2002; Ikeda et al., 2003). Under laboratory conditions *Streptomyces* and other secondary metabolite producers synthesize only a few compounds, whereas more than twenty different secondary metabolite gene clusters are contained within most genomes.

The presence of similar biosynthetic gene clusters in different strains reflects their evolutionary history through vertical as well as horizontal gene transfer from one organism to another, also across species barriers. Individual genes, sub-clusters or whole clusters can be exchanged (Egan et al., 2001; Metsä-Ketelä et al., 2002; Donadio et al., 2005). Consequently, the secondary metabolites from similar clusters may vary as they are often built up of distinct moieties from functional sub-clusters. Therefore, biosynthetic gene clusters are ideal to study evolutionary routes and to gain knowledge of the metabolite’s importance to a particular strain (Fischbach et al., 2008).

The activation of cryptic biosynthetic gene clusters is one main goal in *Streptomyces* research to obtain more and novel bioactive compounds to meet the growing requirements of modern medicine. Potential approaches to successfully activate a gene cluster are summarized in the following paragraph.

The cloning and heterologous expression of complete clusters is one strategy to get access to the genetic potential of *Streptomyces* (Gomez-Escribano and Bibb, 2014). In addition, silent secondary metabolite gene clusters can be activated through genetic manipulation, e.g., by over-expression or deletion of proposed global or specific positive or negative regulatory genes (Makitrynskyy et al., 2013; Gessner et al., 2015). Furthermore, the cultivation of a given strain under different fermentation conditions (Bode et al., 2002) or the co-cultivation with bacterial or fungal strains (Schroeckh et al., 2009) might stimulate the expression of silent clusters.

*Streptomyces diastatochromogenes* Tü6028 is known to produce the antimicrobial compound polyketomycin, a tetracyclic quinone glycoside (Paululat et al., 1999). Recently, we deleted the oxygenase gene *pokOIV* in this strain, resulting in a polyketomycin non-producing mutant (Daum et al., 2009). However, new natural products were synthesized in this mutant. These metabolites were named foxicins A–D. Possibly, foxicins are also produced by the wild type strain but only in little amounts.

**FIGURE 1:**
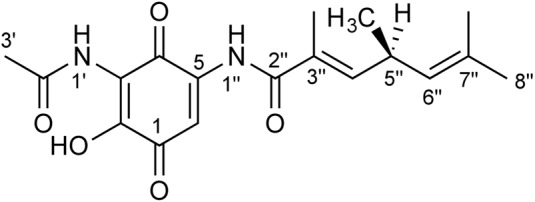
**Structure of foxicin A.** The structure of foxicin A was elucidated as (*S*)-2-hydroxy-3-(acetylamino)-5-(3″, 5″*S*, 7″-trimethyl-hepta-3″*E*, 6″-dienoylamino)-1,4-benzoquinone.

In this study we report on the purification and structural elucidation of foxicin A. The compound consists of an unusual nitrogen-containing quinone moiety linked to a short fatty acid (**Figure 1**).

Furthermore, we identified the corresponding gene cluster, encoding a type I polyketide synthase (PKS I) and an NRPS in the genome of *S. diastatochromogenes* Tü6028. By gene disruption we show that this cluster is responsible for the production of foxicin and we propose the biosynthetic pathway of foxicin A. Similar gene clusters were detected in many other *Streptomyces* genomes. Nevertheless, as no foxicin-like compound has been described so far, we conclude that these clusters are either not expressed or expressed at a very low level under culture conditions.

Foxicin A shows several interesting biological properties: it acts as siderophore binding ferric ions, it shows antibiotic activity and inhibits respiratory electron transfer. The wide distribution of the cluster and the various properties of the compound indicate a major role of foxicin in *Streptomyces* strains.

## Materials and Methods

### Bacterial Culture Condition and Production Analysis

The isolation of polyketomycin of the wild type (wt) strain *S. diastatochromogenes* Tü6028 has been described previously (Paululat et al., 1999). The mutant *S. diastatochromogenes* Δ*pokOIV* (Daum et al., 2009) contains a deletion in *pokOIV*, a gene encoding an oxygenase involved in the biosynthesis of polyketomycin. Mycelium of the mutant strain *S. diastatochromogenes* Δ*pokOIV* was used to inoculate a 300 mL Erlenmeyer flask containing 100 mL of HA medium (yeast extract 0.4%, malt extract 1%, and glucose 0.4% in 1 liter tap water, pH 7.3). The flask was shaken on a rotary shaker (180 rpm) at 28°C. After 48 h, 3 mL of the pre-culture was used to inoculate a second 300 mL Erlenmeyer flask containing 100 mL of HA medium (main culture). After 6 days foxicin A was isolated.

To determine the time dependency of product formation (foxicin A and polyketomycin) the wt strain was grown in 100 mL HA medium for 6 days. Samples were taken after 0, 24, 36, 48, 60, 72, 96, 120, and 144 h of cultivation.

To check the influence of iron on the production of foxicin A, 0.01, 0.1, or 1 mM of FeCl_3_ or FeSO_4_ were added to the production media of *S. diastatochromogenes* Δ*pokOIV*. The experiment was done in triplicate. The strain was incubated for 6 days and the culture was extracted and analyzed by HPLC. For quantification, the integrals of the corresponding chromatogram peaks were compared.

### Isolation of Foxicin A

Mycelium was collected by centrifugation and foxicin A was extracted with acetone (2–3 times the volume of the pellet) by shaking at room temperature for 15 min. After removal of the mycelium by filtration, the extract was evaporated. Finally, this mycelium extract combined with the supernatant was extracted with an equal volume of ethyl acetate. The solvent was removed by evaporation. The crude extract was separated by solid phase chromatography (Oasis HLB 20/35cc) with increasing methanol content (in 10% increments) in the mobile phase. Foxicin A was obtained in the 65 and 70% methanol fraction, foxicin B and C in the 70 and 80% methanol fraction, and foxicin D in the 80% methanol fraction. After thin-layer chromatography in ethyl acetate: formic acid :water (44:3:3) foxicin A (Rf 0.41) was further purified by semi-preparative HPLC (Agilent Technologies), equipped with a Zorbax B-C18 (9.4 × 150 mm) pre-column and Zorbax B-C18 (9.4 mm × 20 mm) main column with acetonitrile + 0.5% acetic acid as buffer A and water + 0.5% acetic acid as buffer B and a flow rate of 2 mL/min. A 3-min washing step with 50% buffer A was followed by a 7-min linear gradient from 50 to 70% of buffer A where the substance was collected. The eluate was dried and resulted in a violet powder. The method was completed by a 4-min delay with 95% buffer A and a 4-min reequilibration step with 50% buffer A.

### Analysis of Foxicin by HPLC/MS

For analysis, HPLC-MS equipped with a XBridge C18 (3.5 μm; 20 mm × 4.6 mm) precolumn and a XBridge C18 (3.5 μm; 100 mm × 4.6 mm) main column, an UV/visible light detector and a mass spectrometer (Agilent, 1100 Series) was used. A flow rate of 0.5 mL/min was used. A 1-min washing step with 20% buffer A was followed by a 7-min linear gradient from 20 to 60% buffer A and a 16-min linear gradient ranges from 60 to 95% buffer A. After a 5-min delay the method completed with a 1-min gradient from 95 to 20% buffer A and a 5-min reequilibration step with 20% buffer A.

### Structure Elucidation by NMR, VCD, and IR Measurements

The exact mass was analyzed on a MAT 95XL-mass spectrometer (Thermo Electron Corporation). ^1^H (600 MHz), ^13^C (150 MHz), and 2D NMR (HSQC, HMBC, ^1^H-^1^H COSY) spectra were carried out on a Varian NMR-S600. Chemical shifts are expressed in δ values (ppm), using the correspondent solvent as internal reference (CDCl_3_: δ_H_ = 7.25, δ_C_ = 77.0 ppm at T = 25°C or DMSO-d_6_: δ_H_ = 2.50, δ_C_ = 39.5 ppm at T = 35°C).

Infrared measurements were carried out on a BRUKER Tensor 27 FT-IR spectrometer equipped with a BRUKER PMA 50 VCD module (Bruker Optik GmbH, Ettlingen). A 100 mM foxicin A solution was prepared in anhydrous CDCl_3_ and placed in a BaF_2_ cell with a path length of 110 μm. Experimental spectra (4 cm^-1^ resolution) represent the average of a 6 h measurement in a rotating cell. IR spectra were corrected by subtraction of the solvent spectrum. VCD spectra were background corrected by solvent subtraction and smoothed by Fourier filtering (8 cm^-1^ resolution). The aperture of the light source was adjusted to a width of 4 mm. Opus 7.0 software (Bruker Corporation) was used to analyze the spectra.

### Conformer Search and Quantum Chemical Calculations

The conformer search was carried out at the MMFF level using Spartan 08 (Wavefunction, Inc., Irvine, CA, USA) and gave a set of 84 possible conformers. The five conformers with the highest population (according to Boltzmann weights calculated in respect to relative energies) account for >99% of the calculated Boltzmann distribution. These conformers were chosen for quantum chemical calculations at the DFT level [B3LYP/6-31+G(d,p)] in Gaussian 09, Revision D.01 (Frisch et al., 2013). All calculations were performed in gas phase, vibrational frequencies were uniformly scaled by an empirical factor of 0.975. Theoretical spectra for each geometry were obtained by adding Lorentzian band shapes (width 6 cm^-1^) to the calculated IR and VCD intensities. The dissymmetry factor spectrum defined as VCD over IR absorbance was obtained with CDSpecTech (Covington and Polavarapu, 2013, 2014).

### Single Crossover of *foxBII* in *S. diastatochromogenes* Δ*pokOIV*

To construct a single crossover of *foxBII* gene (PKS I/NRPS hybrid gene) an internal 2 kb fragment was amplified (primers GCCGGGAAGCTTGTCCTCTTCGCCTC and GTCGTCGGATCCTGCGC CGCCTCGG). The fragment was cloned into pKC1132 (Bierman et al., 1992) at *Hind*III/*Bam*HI cloning site and transferred into *Escherichia coli* ET12567 (*dam*-, *dcm*-, *hsdS*-, cm^r^) (MacNeil et al., 1992), including conjugation plasmid pUZ8002 (Flett et al., 1997). Positive transformants were selected on LB agar supplemented with kanamycin (30 μg mL^-1^) and apramycin (50 μg mL^-1^). The plasmid was transferred into *S. diastatochromogenes* Δ*pokOIV* by conjugation. Exconjugants were incubated on MS media supplemented with apramycin.

### Bioinformatic Analysis of Foxicin Biosynthetic Gene Cluster and Identification of Similar Biosynthetic Gene Cluster

The draft genome of *S. diastatochromogenes* Tü6028 was sequenced at the Centrum of Biotechnology, University of Bielefeld (Greule et al., unpublished). Prediction of the gene clusters was performed using antiSMASH 3.0^1^ (Weber et al., 2015a). The sequence of foxicin 57.6 kb hybrid PKS I/NRPS biosynthetic gene cluster was further analyzed and annotated using BLAST^2^. Similar gene clusters were identified by antiSMASH and BLAST analysis in other *Streptomyces* strains. The genomes of these strains were analyzed individually by the above mentioned programs.

### Phylogenetic Tree of *fox* Homologous Clusters

A phylogenetic tree was calculated by Clustal Omega (Sievers et al., 2011) using Neighbor-Joining method of *foxBII* sequence comparison. For better illustration, the tree is shown without distance correction.

### Siderophore Chrome Azurol S (CAS) – Assay

Chrome azurol S medium was modified after Schwyn and Neilands (1987). For the CAS medium 60.5 mg CAS, 72.9 mg hexadecyltrimetyl ammonium bromide, and 30.24 g piperazine-1,4-bis-(2-ethanesulfonic acid) were dissolved in 990 mL water and mixed with 10 mL iron (III) solution (1 mM FeCl_3_ × 6H_2_O and 10 mM HCl). Purified foxicin A was pipetted to CAS reagent and a color change of the dark blue solution to violet was noted.

### Shift of UV/vis Spectra in the Presence of Iron

Foxicin A was dissolved in MeOH. FeCl_3_ and FeSO_4_ were added in an amount of 0.5 mM to 100 mM. UV/vis spectra were measured by UviLine 9400 spectrophotometer (SI Analytics).

### Isolation of Bacterial Plasma Membranes

*Escherichia coli* BW25113 cells were grown aerobically (180 rpm) at 37°C in baffled flasks using LB-medium. The cells were harvested by centrifugation (5700 × *g*, 10 min, 4°C, Rotor JLA 8.1000, Avanti J-26 XP, Beckman Coulter) in the late exponential phase yielding approximately 6.5 g cells/L. All further steps were carried out at 4°C. After centrifugation, 5 g of the cell pellet were resuspended in fourfold volume of buffer 1 (50 mM MES/NaOH, pH 6.0, 50 mM NaCl, 0.1 mM PMSF supplemented with desoxyribonuclease I) and disrupted by passing twice through a French Pressure Cell Press (110 MPa, SLM-Aminco). Cell debris and non-disrupted cells were removed by centrifugation (9500 × *g*, 20 min, 4°C, Rotor A8.24, RC-5 Superspeed Refrigerated Centrifuge, Sorvall Instruments). The cytoplasmic membranes were obtained from the supernatant by centrifugation at 252000 × *g* (60 min, 4°C, Rotor 70.1Ti, L8-M Ultrafuge, Beckman). The sediment was suspended in an equal volume (1:1, w/v) of buffer 1 and was used directly or frozen in liquid nitrogen and stored at -80°C.

### Determination of NADH Oxidase Activity

The NADH oxidase activity of cytoplasmic membranes was measured with a Clark-type oxygen electrode (RE K1-1, Oxytec) at 30°C. To calibrate the electrode 2 mL 50 mM MES/NaOH, pH 6.0, 50 mM NaCl, 5 mM MgCl_2_ were deoxygenized by adding sodium dithionite and the signal was set to 237 μM oxygen (Weiss, 1970). Each measurement was performed with 2 mL buffer containing 5 μL of the membrane suspension at 30°C. The reaction was started by adding 1.25 mM NADH. 50–500 μM foxicin A was added to the assay to test its inhibitory action on cell respiration.

### Agar Diffusion Assay

Antimicrobial activity of foxicin A was determined by the agar plate diffusion method on a paper disk (6 mm diameter). 100 μg per disk of foxicin A were tested against *Streptomyces viridochromogenes* Tü57 (Hütter, 1962), *Actinokineospora bangkokensis* (Intra et al., 2013), *Saccharothrix espanaensis* (Kong et al., 1998), *Bacillus subtilis* (Ehrenberg) COHN ATCC6051 (American Type Culture Collection, 18th Edition, 1992), *E. coli* XL1-Blue, *Fusarium verticillioides* DSM 62264, *Candida parapsilosis* DSM 5784, *Synechococcus* sp. PCC7002 and *Synechocystis* sp. PCC6803, *Mycobacterium smegmatis* mc^2^ 155. The compound was dissolved in methanol. After evaporation of the methanol, disks were fixed on the test-plates and incubated over night at 30 or 37°C. Polyketomycin, apramycin, and methanol were used as controls. The following media were used for the assay: LB-medium (tryptone 1%, yeast extract 0.5%, NaCl 0.5%) for *E. coli* and *Bacillus*, MS medium (soya flour 2%, mannitol 2%) for actinomycetes strains, BG11-medium (NaNO_3_ 0,15%, K_2_HPO_4_ 0.004%, MgSO_4_ × 7H_2_O 0.0075%, CaCl_2_ × 2H_2_O 0.0036%, citric acid 0.0006%, ammonium ferric citrate 0.0006%, EDTA 0.0001%, Na_2_CO_3_ 0.002%, trace elements 1.0 mL) for cyanobacterial strains, PDA-medium (potato extract 0,4%, dextrose 2%) for *Fusarium*, YPD-medium (yeast extract 1%, peptone 2%, glucose 2%) for *Candida* and Middlebrook 7H9 Broth (7H9 broth base 0.52%, 40% glycerol 5.5 mL) for *Mycobacterium*. For agar plates 2% agar-agar was added to the media.

To assay the herbicidal property, 0.1, 1, and 5 μM of foxicin A was added to 6 mg of *Arabidopsis thaliana* wt ecotype Wassilewskija seeds that were plated onto plates. The plants were grown for 2 weeks in a phytochamber with long-day conditions (16/8 h), 100 μE m^-2^ s^-1^ light intensity and 25°C constant temperature.

To check for H_2_O_2_ sensitivity, 2, 5, and 10 μL of 5% H_2_O_2_ solution were pipetted on paper disks (6 mm diameter) and placed on MS and TSB culture plates of *S. diastatochromogenes* WT, Δ*pokOIV* mutant and Δ*pokOIV*/*foxBII*::pKC1132 mutant. The strains were incubated at 28°C for 5 days and inhibition zone was measured.

### Cell Viability Assay (MTT-Assay)

The effect on cell viability of foxicin A was tested against cancer cell lines CCRF-CEM, CEM-ADR5000 and Jurkat cells and peripheral blood mononuclear cells (PBMC) using the MTT assay as previously described (Calderón et al., 2014). In brief, cells were seeded in 96-well plates at a density of 4 × 10^4^ cells/well and incubated for 24 h with various concentrations of foxicin A. The chemotherapeutic agent doxorubicin was used as a positive control, and DMSO (0.1%) was the solvent control. The data are expressed as the mean ± SD of three independent experiments.

## Results

### Production and Isolation of Foxicin A from *S. diastatochromogenes* Tü6028

**FIGURE 2:**
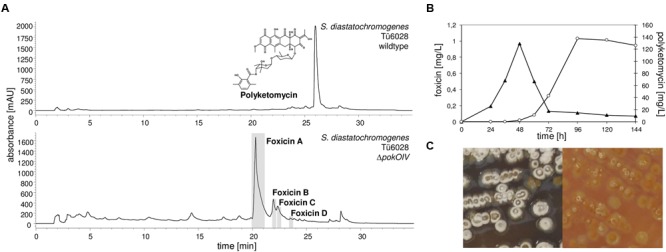
**Production of polyketomycin and foxicin in *Streptomyces diastatochromogenes* Tü6028. (A)** HPLC chromatogram of *S. diastatochromogenes* Tü6028 wild type at λ430 nm with polyketomycin structure (top) and of Δ*pokOIV* mutant at λ320 nm (below); **(B)** Production of foxicin A (▴) and polyketomycin (O) in *S. diastatochromogenes* Tü6028 wild type; **(C)** Morphology of *S. diastatochromogenes* Tü6028 wild type (left) and Δ*pokOIV* mutant (right). On the plate, the Δ*pokOIV* mutant appears yellow and deficient in producing spores and melanins (dark color).

*Streptomyces diastatochromogenes* Tü6028 is known to synthesize polyketomycin, which is produced at high levels after 96 h of cultivation in HA medium. During our studies on polyketomycin biosynthesis we deleted the oxygenase gene *pokOIV* (Daum et al., 2009). The mutant failed to produce polyketomycin, instead we observed the accumulation of novel compounds, which we named foxicin A, B, C, and D (minor compounds) (**Figure 2A**). A careful analysis of extracts of the wild type strain showed that foxicins are also produced, but with significantly lower titers. Foxicin production in the wt strain reaches a maximum after 48 h of incubation (**Figure 2B**). After 96 h less than 5% of the initial foxicin A concentration was detected. In *S. diastatochromogenes* Δ*pokOIV*, foxicin production reaches its maximum after 6 days. The mutant also produces fewer spores and visibly lower amounts of melanin, as indicated by the color of the cultivation medium (**Figure 2C**). Cultivation of *S. diastatochromogenes* Δ*pokOIV* in 5 L of HA production medium yielded 9.8 mg of foxicin A (1.96 mg/L) and 3.4 mg of foxicin B (0.68 mg/L), and even lower amounts of foxicins C and D.

### Physiocochemical Properties and Structure Elucidation of Foxicin A

The physicochemical properties of foxicin A are summarized in Supplementary Table S1. Foxicin A is soluble in common organic solvents such as MeOH, CH_3_CN, CHCl_3_ and DMSO, but is insoluble in H_2_O. HR-ESI-MS analysis (*m/z* found: 346.1532; calculated: 346.1529) revealed the molecular formula as C_18_H_22_N_2_O_5_. The UV/vis light spectra of the foxicin derivates A–D are similar (Supplementary Figure S1). Foxicin A shows absorption maxima at 246, 280, 316, 390 and a small peak at 462 nm.

**FIGURE 3:**
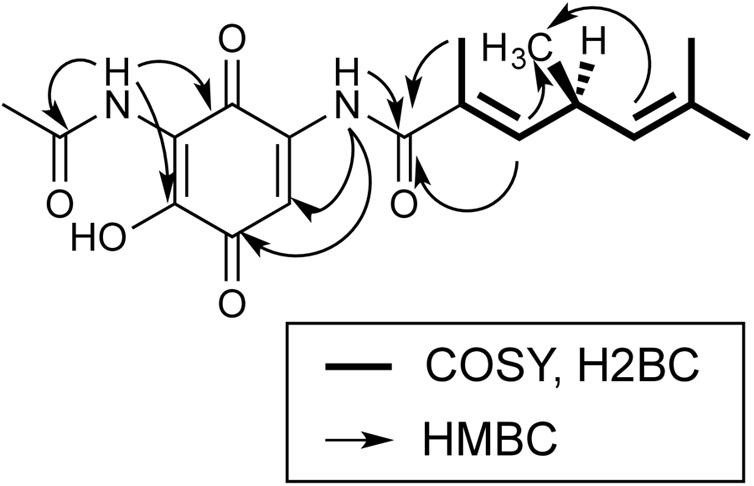
**Important NMR correlations of foxicin A**.

NMR-data of foxicin A were recorded in CDCl_3_ and DMSO-d_6_. In Supplementary Tables S2 and S3, NMR assignments of 1D NMR (^1^H, ^13^C) and 2D NMR (^1^H-^1^H-COSY, HSQC, H2BC, and HMBC) experiments are summarized and Supplementary Figures S2–S14 show the respective spectra. The ^13^C NMR spectrum shows 18 carbon signals, which could be assigned to five methyl, three methine groups and nine quarternary carbon atoms by the use of HSQC. A 1,4-benzoquinone system was identified from typical carbonyl chemical shifts (*δ*_C_ = 182.3 and *δ*_C_ = 178.6 ppm). The benzoquinone is substituted with a hydroxyl group in position C-2 and an amino-acetate at position C-3, as established by HMBC correlations C-2/6-H, C-2/1′-NH, and C-4/1′-NH. Moreover, an additional side chain is attached at C-5 via an amide functionality, as proven by the HMBC signals C-4/1″-NH and C-6/1″-NH. The side chain contains two double bonds, which are both trisubstituted. One double bond is in conjugation to the amide carbonyl as proven by the HMBC signal C-2″/4″-H and bears a methyl group at C-3″ as shown by the HMBC cross peaks C-2″/3″-CH_3_, 3″-CH_3_/4″-H, this double bond is in *E* configuration as proven by the ROESY signal 3″-CH_3_/5″-H. The second double bond was determined as a C-6″ = C-7″ double bond with two methyl groups at C-7″ based on HMBC correlations. The two double bonds are connected via the C-5 methine group, which is methyl substituted as established by COSY couplings 4″-H/5″-H, 5″-H/6″-H and H2BC signals C″-5/4″-H and C-5″/6″-H. The important 2D correlations are shown in **Figure 3**.

**FIGURE 4:**
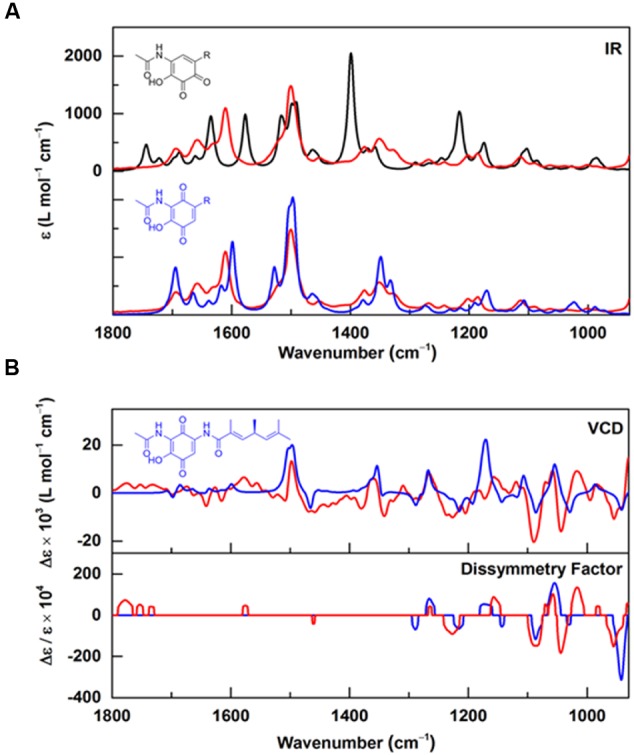
**Infrared and VCD spectra of foxicin A. (A)** Experimental IR spectra recorded for foxicin A (red) overlayed with IR spectra calculated at the B3LYP/6-31+G(d,p) level for the *ortho*-quinone (black) and for the *para*-quinone (blue) with considerably better agreement for *para* (spectra are offset for better comparison); **(B)** Experimental (red) VCD and dissymmetry factor spectra in comparison to VCD and dissymmetry factor spectra calculated for (*S*)-foxicin (blue). The good overall agreement allows for assignment of the *S*-configuration.

Based on our 1- and 2D NMR data, the absolute configuration of foxicin A could not be solved unambiguously. Therefore, we analyzed this compound by comparing vibrational circular dichroism (VCD) and infrared (IR) spectra to spectra from quantum chemical calculations. A molecular model of foxicin A was constructed and subjected to a conformer search algorithm employing molecular mechanics (MMFF). The conformer models were then subjected to a geometry optimization at the DFT level [B3LYP/6-31+G(d,p)] and the relative energies and IR absorbance and VCD intensities were calculated. The comparison of the Boltzmann-weighted average of the spectra calculated for five conformers of foxicin A and the experimental VCD spectrum (**Figure 4**) allowed for the assignment of the absolute conformation as well as the configuration of foxicin A as (*S*)-2-hydroxy-3-(acetylamino)-5-(3″, 5″*S*, 7″-trimethyl-hepta-3″*E*, 6″-dienoylamino)-1,4-benzoquinone. The structure of foxicin A is shown in **Figure 1**.

Foxicin B has the same mass (346 g/mol) as foxicin A. Foxicin C and foxicin D are only produced in low amounts. Both compounds have a molecular weight of *M* = 360 g/mol, based on the deprotonated molecular ion peak at *m/z* 359.2 in the negative ion mode CI-MS spectrum.

### Identification and Sequence Analysis of the Biosynthetic Gene Cluster

**FIGURE 5:**
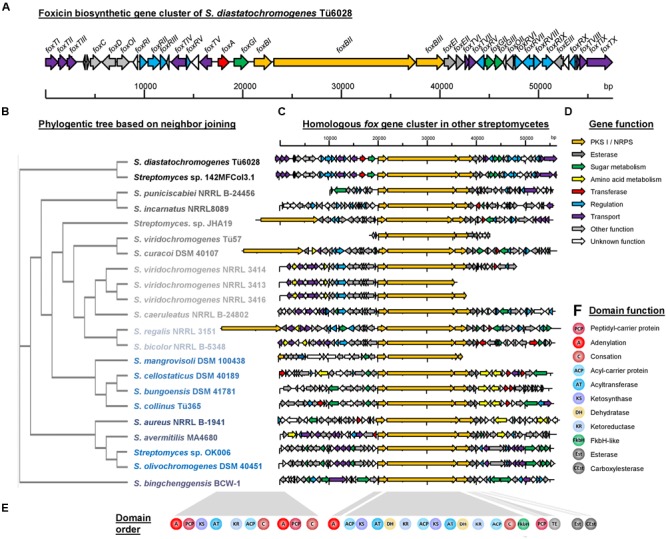
**Foxicin biosynthetic gene clusters in different *Streptomyces* strains. (A)** Organization of the foxicin biosynthetic gene cluster in *S. diastatochromogenes* Tü6028. The *fox* gene cluster spans 57.6 kb and contains 41 ORFs. Genes are indicated by different arrowheads according to their proposed function. **(B)** Phylogenetic tree based on Neighbor Joining without distance corrections of sequence comparison of *foxBII*, **(C)** homologous *fox* gene clusters in other *Streptomyces* strains, **(D)** predicted gene function and **(E)** domain assembly line of biosynthesis genes and **(F)** predicted domain function.

Due to its chemical structure, foxicin A is likely to be a product of a type I polyketide synthase (PKSI) and a non-ribosomal peptide synthetase (NRPS). Bioinformatics analysis of the 7.9 Mb draft genome sequence of *S. diastatochromogenes* Tü6028 revealed 23 putative secondary metabolite gene clusters, but only one cluster containing genes with both PKSI- and NRPS function. The cluster was assigned to 57.6 kb with an overall GC content of 72.4%. The annotation analysis revealed 41 open reading frames (ORFs) putatively involved in foxicin A-D biosynthesis (**Table 1**). The genetic organization of the biosynthetic gene cluster (*fox* gene cluster) is shown in **Figure 5A**. The GenBank accession number of the nucleotide sequence is KT440882. In order to verify the correct assignment of the *fox* gene cluster, we constructed the inactivation plasmid pKC1132_SC_foxBII containing a 2 kb homologous region of *foxBII*. Conjugation between *E. coli* ET12567 and *S. diastatochromogenes* Tü6028 Δ*pokOIV* and integration of the plasmid into *foxBII* by single crossover recombination resulted in apramycin-resistant mutants. Integration of the plasmid into *foxBII* by single crossover recombination was confirmed by PCR. Loss of ability of the mutant strain to produce foxicins confirmed the correct assignment of the *fox* gene cluster.

**Table 1:** Proposed functions of open reading frames in the foxicin biosynthetic gene cluster.

ORF	aa	Proposed function
*foxTI*	422	ABC transporter, substrate binding
*foxTII*	304	ABC transporter, permease
*foxTIII*	309	ABC transporter, permease
*foxHI*	66	Hypothetical protein
*foxHII*	65	Hypothetical protein
*foxC*	346	ATP/GTP binding protein
*foxD*	451	Dipeptidase / deacetylase
*foxOI*	409	Hydrogenase
*foxHIII*	153	Hypothetical protein
*foxRI*	237	TetR transcriptional regulator
*foxRII*	417	Two-component system sensor kinase
*foxRIII*	221	Two-component system response regulator
*foxHIV*	81	Hypothetical protein
*foxTIV*	493	Transmembrane efflux protein
*foxRIV*	137	MarR transcriptional regulator
*foxHV*	246	Hypothetical protein
*foxTV*	399	Integral membrane transport protein
*foxA*	371	Acetyl transferase
*foxGI*	481	Glyceraldehyde-3-phosphate dehydrogenase
*foxBI*	583	Non-ribosomal peptide synthetase (A-domain)
*foxBII*	4807	Modular polyketide synthase (ACP-KS-AT-DH^∗^-KR-ACP-KS-AT-DH-KR-ACP), non-ribosomal peptide synthetase (C-domain), FkbH
*foxBIII*	945	Non-ribosomal peptide synthetase (PCP-TE)
*foxEI*	378	Esterase
*foxEII*	259	Carboxylesterase
*foxTVI*	131	Drug resistance transporter
*foxTVII*	253	Drug resistance transporter
*foxRV*	261	Regulator
*foxGII*	306	Sugar isomerase
*foxGIII*	283	Sugar-bisphosphate aldolase
*foxHVI*	99	Hypothetical protein
*foxOII*	259	Dioxygenase
*foxRVI*	257	AraC family transcriptional regulator
*foxRVII*	380	rRNA methyltransferase
*foxRVIII*	241	Alkylated DNA repair protein
*foxRIX*	371	ROK family transcriptional regulator
*foxEIII*	310	Phosphotriesterase
*foxHVII*	194	Hypothetical protein
*foxRX*	191	TetR family transcriptional regulator
*foxTVIII*	101	Membrane protein
*foxTIX*	252	ABC transporter, ATP-binding protein
*foxTX*	853	ABC transporter, substrate-binding protein

### Characterization of the Deduced Amino Acid Sequences and Putative Model of the Biosynthesis of Foxicins

Based on bioinformatics analysis of the *fox* gene cluster of *S. diastatochromogenes* Tü6028, a putative biosynthetic pathway for foxicins’ biosynthesis is proposed. The 14.4 kb gene *foxBII* encodes a protein with predicted modular type I polyketide synthase function, with one loading module, two extender modules, a FkbH-like domain, and one condensation domain (C) of a NRPS. The loading module of FoxBII contains only one ACP. Based on the structure of foxicin A we propose isobutyryl-CoA as being the respective starter unit. Module I and II consist of a ketosynthase (KS), acyltransferase (AT), dehydratase (DH), ketoreductase (KR) and an ACP domain, respectively. Both ATs show specificity for methylmalonyl-CoA as predicted using antiSMASH (Weber et al., 2015a), which is in line with the structure of foxicin A.

Foxicin A possesses a double bond between the starter unit and the first extender unit in *β,γ*-position. Similar structural elements are known from ansamitocin-, rhizoxin-, bacillaen and corallopyronin. It has been shown that special dehydratases or additional shift modules are responsible for the double bond shift in *β,γ*-position during the biosynthesis of these molecules (Taft et al., 2009; Kusebauch et al., 2010; Moldenhauer et al., 2010; Lohr et al., 2013). In some DH domains, the conserved motif is mutated. The DH domain of module I of the *fox* cluster shows in contrast to other DH domains a motif of HxxxGxxxxS instead of the conserved HxxxGxxxxP motif, indicating that this domain could introduce the *β,γ*-double bond during foxicins’ biosynthesis.

The FkbH-like domain is likely to incorporate a glyceryl moiety (Chan et al., 2006; Dorrestein et al., 2006; Sun et al., 2008). The three genes *foxGI, foxGII* and *foxGIII*, also located in the *fox* biosynthetic gene cluster, encode for enzymes known to be involved in sugar metabolism. The proposed functions of the enzymes are glyceraldehyde-3-phosphate dehydrogenase, sugar isomerase and aldolase. Most likely the three of them provide bisphosphoglycerate, which is transferred onto the carrier protein of FoxBIII.

The adenylation domain (A) might be encoded by *foxBI*, which is separated from *foxBIII* by the PKS gene *foxBII*. *In silico* analysis did not indicate an A domain specificity, but based on the structure of foxicin A, a non-proteinogenic amino acid with two amide groups similar to 2,4-diamino-3-oxobutanoic acid might play a role. This moiety is then most likely linked to the glyceryl-CP.

**FIGURE 6:**
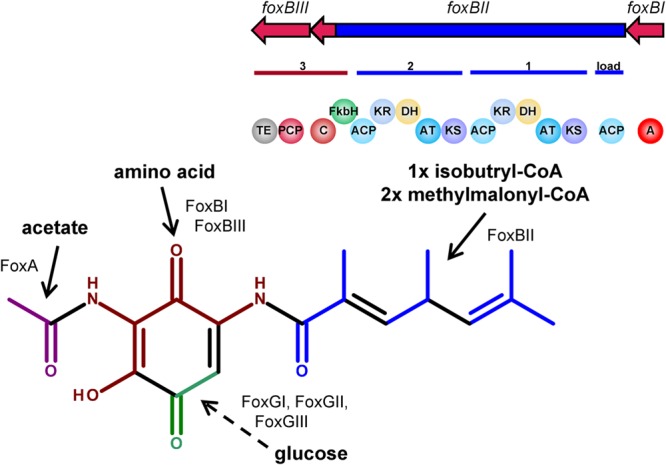
**Proposed biosynthetic pathway of foxicin.** FoxBII is proposed to be involved in the formation of the polyketide side chain (blue), FoxBI and FoxBIII in the incorporation of an amino acid (dark red) and FoxGI-III with FkbH domain of FoxBII in the incorporation of a C2 sugar moiety (green). N-acetylation might be catalyzed by FoxA (magenta).

We propose that the biosynthesis starts with the PKS I of FoxBII and the generated polyketide chain is then transferred onto the amino acid bound to the glyceryl-CP of FoxBIII. Finally, the molecule is cleaved from the enzyme by the thioesterase domain (TE) of FoxBIII, followed by the ring formation. The responsible enzyme for the ring formation is unknown, but with BLAST analysis we identified candidate genes as *foxEI* or *foxEII*, with proposed esterase activity. *N*-acetylation catalyzed by FoxA results ultimately in foxicin A (**Figure 6**). After cleavage from the enzyme complex foxicin A gets further modified.

At each end of the *fox* gene cluster two ABC transporter genes are located (*foxTI*-*TIII* and *foxTVIII*-*X*). In addition, *foxTIV* encodes a transmembrane efflux protein, *foxTV* an integral membrane transport protein, *foxTVI* a transporter belonging to the EmrB/QacA family and *foxTVII*, an export protein. The biosynthetic gene cluster of foxicins also includes 10 regulatory proteins (FoxRI-FoxRX), indicating a complex regulation of the outlined biosynthesis.

### Similar *fox* Biosynthetic Gene Clusters in Various *Streptomyces* Strains

Similar *fox* gene clusters were identified in the genomes of 21 additional *Streptomyces* strains (**Figures 5C,D**): *S. aureus* NRRL B-1941 (Doroghazi et al., 2014), *S. avermitilis* MA4680 (Ikeda et al., 2003), *S. bicolor* NRRL B-5348 (Doroghazi et al., 2014), *S. bingchenggensis* BCW-1 (Wang et al., 2010), *S. bungoensis* DSM 41781, *S. caeruleatus* NRRL B-24802, *S. cellostaticus* DSM 40189, *S. collinus* Tü365 (Rückert et al., 2013), *S. curacoi* DSM 40107, *S. incarnatus* NRRL 8089 (Oshima et al., 2015), *S. mangrovisoli* DSM 100438, *S. olivochromogenes* DSM 40451, *S. puniciscabiei* NRRL B-24456, *S. regalis* NRRL 3151, *S. viridochromogenes* NRRL 3414, NRRL 3416 and NRRL_3413, *S. viridochromogenes* Tü57 (Grüning et al., 2013), *Streptomyces* sp. 142MFCol3.1, *Streptomyces* sp. JHA19 (Matsunaga et al., 2015) and *Streptomyces* sp. OK006 (Klingeman et al., 2015). In these strains homologs of *foxBI, foxBII, foxBIII, foxEI*, and *foxEII* are located next to each other with high sequence identities of 73% up to 96% (Supplementary Table S4). The closest homologous cluster is located in *Streptomyces* sp. 142MFCol3.1. A phylogenetic tree based on *foxBII* sequence analysis is shown in **Figure 5B**. In all strains the NRPS/PKS I hydride enzyme complex (*foxBI* – *foxBIII*) consists of the same series of catalytic domains ([A] – [ACP-KS-AT-DH-KR-ACP-KS-AT-DH-KR-ACP-C-FkbH] – [PCP-TE]) (**Figures 5E,F**). The strains *Streptomyces* sp. JHA19, *S. curacoi* and *S. regalis* have an additional PKS/NRPS hybrid gene upstream of *foxBI-III* with [A-PCP-KS-AT-KR-ACP-C-A-PCP-C] catalytic domains. The phylogenetic tree that is based on *foxBII* illustrates that the biosynthetic gene clusters of these strains do not originate from one clade.

In most of the strains *foxEI* and *foxEII* reside adjacent to *foxBI*-*foxBIII* indicating that they might play an important role in the biosynthesis of these secondary metabolites. Additionally, in most of the clusters genes from sugar and amino acid metabolism, as well as methyl-/acetyl transferases were identified.

The described genes in the predicted *fox* clusters do not agree in detail, therefore we assume that the corresponding compounds might exhibit structural differences. Considering the discrete array of catalytic domains, however, we expect similar polyketide tails in all foxicins A–D-like substances. To the best of our knowledge, none of the mentioned strains synthesizes a compound related to foxicins. The responsible gene clusters seem to be silent or have, until now, not been studied. In Supplementary Table S4, an overview of the described *Streptomyces* strains is shown, as well as their known secondary metabolites and the percentage identity of the identified genes with *foxBI, foxBII, foxBIII, foxEI*, and *foxEII*.

### Activity of Foxicin A

**FIGURE 7:**
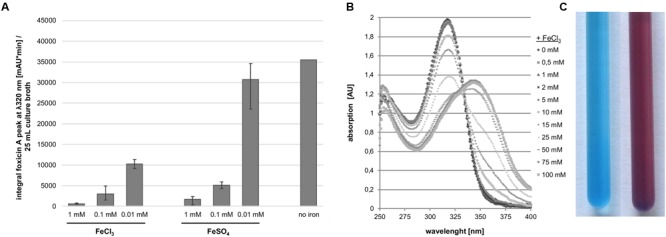
**Interaction of foxicin with ferric ions. (A)** Foxicin production with supplementation of different amounts of ferric ion, calculated integral of the foxicin A peak at 320 nm/25 mL culture; **(B)** Shift of UV/vis spectrum of foxicin A in presence of FeCl_3_; **(C)** CAS-assay of foxicin A, color exchange from blue (left) to violet (right).

In order to understand the function of foxicin A we attempted to characterize this molecule in greater detail. By means of LC-MS analysis we could show that Foxicin A production in *S. diastatochromogenes* is inhibited by ferric ions supplemented to the growth medium. The supplementation of either 1 mM FeCl_3_ or FeSO_4_ to the production medium led to a more than 75-fold decrease in foxicin A formation in *S. diastatochromogenes* Δ*pokOIV* (**Figure 7A**). The addition of FeCl_3_ to foxicin A resulted in a shift of the UV/vis maximum at 320–350 nm (**Figure 7B**). Using the CAS assay (Schwyn and Neilands, 1987), we observed a color change from blue to violet (**Figure 7C**), indicating that foxicin A acts as a siderophore.

As foxicin A contains a quinone moiety similar to molecules involved in various redox processes within the cell (Brunmark and Cadenas, 1989), the compound was tested for its property as an electron acceptor or inhibitor of the respiratory chains. Foxicin A was added to *E. coli* cytoplasmic membranes and the respiratory chain activity was measured with a Clark-type electrode by starting the reaction with NADH as electron donor. 500 μM foxicin A blocked O_2_ consumption to 30% indicating its role as a weak inhibitor of the electron transfer chain (Supplementary Table S5). In contrast to other quinones (Kawamukai, 2002) foxicin A does not protect cells from oxidative stress induced by H_2_O_2_ (Supplementary Figure S15).

To identify the antibiotic potential of foxicin A, an inhibition zone test was conducted. Therefore, 100 μg foxicin A were applied to paper disks that were transferred to different culture plates. The compound showed moderate activity against *Streptomyces viridochromogenes, Saccharothrix espanaensis* and the cyanobacterial strains *Synechococcus* sp. and *Synechocystis* sp. The tested amount of foxicin A did not visibly influence the growth of the bacterial strains *E. coli* XL1-Blue, *Bacillus subtilis, Mycobacterium smegmatis*, the fungal strains *Candida parapsilosis* and *Fusarium verticillioides* (Supplementary Table S6) and the plant *Arabidopsis thaliana.* To determine the effect of foxicin A on cell viability of human cells, leukemia cell lines CCRF-CEM, CEM-ADR5000 and Jurkat cells as well as non-cancer PBMCs were stimulated for 24 h with foxicin A at a concentration range of 0.6–80 μM. However, foxicin A showed no significant effect on cell viability, as measured by using the MTT assay (Supplementary Figure S16).

## Discussion

As a result of the deletion of the structural gene *pokOIV* of polyketomycin biosynthesis, we identified novel secondary metabolites in *S. diastatochromogenes* Tü6028, named foxicins. In the wild type strain, the gene cluster is expressed only at low levels. In contrast, the Δ*pokOIV* mutant produces higher amounts of the foxicin derivatives A–D, enabling further investigations of these fascinating compounds. Noteworthy, the mutant strain produces less spores and melanin, indicating a crucial role of polyketomycin for the differentiation and stress response.

The structure of foxicin A could not be completely elucidated by NMR analysis. Eventually, the combination of NMR, IR, and VCD spectra analysis led to the solution of the structure of foxicin A. Foxicin A shows several unusual features. It has a *para*-quinone moiety, with two amide groups on each site, one is further acetylated and a short fatty acid side chain with non-conjugated double bonds.

The structure of the compound and the encoding genes in the *fox* cluster suggest a novel biosynthetic pathway. We conclude that foxicins are hybrid compounds with structural elements most probably derived from an amino acid, a C2-moiety derived from sugar metabolism, a polyketide chain of isobutyryl-CoA and methylmalonyl-CoA, and *N*-acetylation. Although aminoquinones are common structural elements in natural products, none of those that are known is similar to foxicin. Compounds such as *N*-(3-carboxylpropyl)-5-amino-2-hydroxy-3-tridecyl-1,4-*benzo*quinone from plant roots of *Embelia ribes* (Lin et al., 2006), nakijiquinones A-I from marine sponges (Shigemori et al., 1994; Kobayashi et al., 1995; Takahashi et al., 2008, 2009), actinomycin of different *Streptomyces* strains (Waksman and Gregory, 1954) and the large group of ansamycins (Brufani et al., 1973; Oppolzer and Prelog, 1973; White et al., 1973; Rinehart and Shield, 1976) and mitomycin/porfiromycin (Webb et al., 1962), comprise a 3-amino-1,4-*benzo*quinone moiety. In few cases, aminoquinones are substituted with an additional amino group such as the *Streptomyces* products abenquines A–D (Schulz et al., 2011), the antitumor compounds streptonigrin (Rao and Cullen, 1959–1960) and lavendamycin (Doyle et al., 1981), or the fungal pigments lepiotaquinone (Spiteller et al., 2003) or lilacinone (Aulinger et al., 2000) and aminoglycoside antibiotics such as streptomycin. For many of these products, it was shown that the aminoquinone moiety was built by either amination of glucose-6-phosphate (Llewellyn and Spencer, 2006) or the aminoshikimate pathway (Floss et al., 2011).

Quinone moieties are commonly found in natural products. One major group are the ubiquinones, for example, being important electron carriers in respiration and photosynthesis. Furthermore, they are involved in all kinds of redox reactions and play a crucial role as antioxidants (Kawamukai, 2002). Their long isoprenoid chains lead to the ability to penetrate biological membranes. The short polyketide tail of foxicin A indicates that the compound is not located in the membrane. Idebenone, a synthetic quinone with similarities to ubiquinones, but with a much shorter, less lipophilic tail is predominantly active in the cytoplasm and not in cellular membranes. It is a potent antioxidant, prevents lipid peroxidation and protects against ROS-induced damage in multiple systems (Suno and Nagaoka, 1984; Sugiyama et al., 1985; Rauchová et al., 2006). Foxicin A has even a shorter chain than idebenone. The presence of foxicin A in the culture medium supports the assumption that foxicin A activity is not associated with membrane binding.

Like other quinones, foxicin A is able to accept electrons, but does not act as an antioxidant. *In vitro*, foxicin A inhibits respiratory function in *E. coli* membranes, without affecting the *in vivo* viability of the strain. Foxicin A obstructs the growth of other actinomycetes strains, as well as cyanobacterial species. In contrast, the molecule does not visibly influence the growth of *Arabidopsis thaliana*. Therefore, it may also interact with the photosynthesis machinery, but cannot pass through all types of cell walls. Further studies are needed to support this hypothesis.

The main function of the foxicins is most likely explained by their ability to act as a siderophore. Surprisingly, foxicin A does not inhibit the viability of human cells, even though it is able to bind ions from the medium. Ions are essential for all organisms. The lack of ions, especially ferric ions, often limits the growth of bacteria in their natural habitat. Therefore, siderophores are vital molecules that are released into the medium, and, after scavenging ions, are actively transported back into the cell. The strain *S. diastatochromogenes* Δ*pokOIV* produces less foxicins in the presence of ferric ions in the production media. Foxicin A interacts directly with iron as shown by the CAS assay and a shift of the absorbance maximum in the UV/vis spectrum.

Investigations on siderophores are nowadays in the focus of many research groups, in order to obtain new antibacterial compounds, by employing the ‘trojan horse’ strategy. The outer membrane is an important barrier of Gram-negative bacteria, as well as of mycobacteria. Diarra et al. (1996) and Möllmann et al. (2009) have intriguingly shown that the linkage of an antibiotic to a siderophore can lead to facilitated transport *via* specific transporters into the cell with subsequent death of the pathogens.

The siderophore yersiniabactin has been found in the plague bacterium *Yersinia* (Pelludat et al., 1998) and in other bacteria such as the nematode symbiont *Photorhabdus luminescens* (Duchaud et al., 2003), the plant pathogen *Pseudomonas syringae*, pathogenic strains of *E. coli* (Bultreys et al., 2006) and even in the Gram-positive marine bacterium *Salinispora tropica* (Udwary et al., 2007). The ability to synthesize siderophores gives special benefits to a strain and the evolutionary driving force to keep the biosynthetic gene clusters. The foxicin cluster was identified in more than twenty additional *Streptomyces* strains, which were isolated at different places around the world, indicating an evolutionary early origin. Until now (11/2016) genome sequences of 844 *Streptomyces* strains are available on NCBI. This means that the cluster is present in about 2.6% of all *Streptomyces* strains. It is anticipated that further genome sequencing of many more *Streptomyces* strains will reveal additional homologous *fox* biosynthetic gene clusters. Surprisingly, the *fox* cluster was not detected in any other actinomycetes genus. Although horizontal gene transfer is common in the *Streptomyces* genus, it is unexpected that the cluster is found in that many other strains. In addition, the organization of the structural genes *foxBI, foxBII, foxBIII, foxEI*, and *foxEII* remained the same with high sequence similarity. Therefore, we assume that the respective products should only by slightly different to foxicin A-D. In the three strains comprising of additional PKS/NRPS genes, the product might be more complex, e.g., possessing a second polyketide chain. Many of the investigated strains are known producers of secondary metabolites, but not of compounds similar to foxicins. Therefore, the identified clusters seem to be silent in these strains or the compound is produced only in very little amounts. As a consequence, its presence could have been overlooked in routine natural compound screening, as it had been the case for polyketomycin. The high similarity of the clusters indicates an evolutionary driving force to keep the biosynthetic gene clusters in place and consequently a major role of the compounds for *Streptomyces* strains.

## Author Contributions

AG designed and performed experiments, analyzed data, proposed the biosynthesis model and wrote the paper; AG, IP, and SZ purified foxicin A; DD performed CAS assay; MaM and SL conducted and analyzed VCD and IR measurements; SZ, CJ-T, TP, S-ML, MM, and PB interpreted NMR data, SB performed *in vitro* respiratory assay; CDF conducted MTT assay; AB, IM, and TF administered the experiments; all authors have given approval to the final version of the manuscript.

## Conflict of Interest Statement

The authors declare that the research was conducted in the absence of any commercial or financial relationships that could be construed as a potential conflict of interest.

## Footnotes

1 http://antismash.secondarymetabolites.org/

2 http://blast.ncbi.nlm.nih.gov

## Abbreviations

A domain: adenylation domain
ACP: acyl carrier protein
C domain: condensation domain
CAS: chrome azurol S
DFT: density functional theory
DH: dehydratase
EtOAc: ethyl acetate
IR: infrared
KR: ketoreductase
KS: ketosynthase
Mbp: mega base pairs
mMFF: molecular mechanics
MTT: 3-(4,5-dimethylthiazol-2-yl)-2,5-diphenyl tetrazolium bromide
NRPS: non-ribosomal peptide synthetase
PCP: peptidyl carrier protein
PKS: polyketide synthase
VCD: vibrational circular dichroism
wt: wild type

## References

[B1] Aulinger K. Arnold N. Steglich W. (2000). Metabolites of 2-aminophenol from fruit bodies of *Lepiota americana* (Agaricales). *J. Biosci.* 55 481–484. 10.1515/znc-2000-5-628 10928564

[B2] Bentley S. D. Chater K. F. Cerdeño-Tárraga A.-M. Challis G. L. Thomson N. R. James K. D. (2002). Complete genome sequence of the model actinomycete *Streptomyces coelicolor* A3(2). *Nature* 417 141–147. 10.1038/417141a 12000953

[B3] Bérdy J. (2012). Thoughts and facts about antibiotics: Where we are now and where we are heading. *J. Antibiot. (Tokyo)* 65 441–441. 10.1038/ja.2012.54 22922479

[B4] Bierman M. Logan R. O’Brien K. Seno E. T. Rao R. N. Schoner B. E. (1992). Plasmid cloning vectors for the conjugal transfer of DNA from *Escherichia coli* to *Streptomyces* spp. *Gene* 116 43–49. 10.1016/0378-1119(92)90627-2 1628843

[B5] Bode H. B. Bethe B. Höfs R. Zeeck A. (2002). Big effects from small changes: possible ways to explore nature’s chemical diversity. *Chembiochem* 3 619–627. 12324995 10.1002/1439-7633(20020703)3:7<619::AID-CBIC619>3.0.CO;2-9

[B6] Brufani M. Kluepfel D. Lancini G. C. Leitich J. Mesentsev A. S. Prelog V. (1973). The biogenesis of rifamycin S. *Helv. Chim. Acta* 56 2315–2323. 10.1002/hlca.19730560718 4761273

[B7] Brunmark A. Cadenas E. (1989). Redox and addition chemistry of quinoid compounds and its biological implications. *Free Radic. Biol. Med.* 7 435–477. 10.1016/0891-5849(89)90126-3 2691341

[B8] Bultreys A. Gheysen I. de Hoffmann E. (2006). Yersiniabactin production by *Pseudomonas syringae* and *Escherichia coli*, and description of a second yersiniabactin locus evolutionary group. *Appl. Environ. Microbiol.* 72 3814–3825. 10.1128/AEM.00119-06 16751485 PMC1489633

[B9] Calderón C. De Ford C. Castro V. Merfort I. Murillo R. (2014). Cytotoxic clerodane diterpenes from *Zuelania guidonia*. *J. Nat. Prod.* 77 455–463. 10.1021/np400672g 24484281

[B10] Chan Y. A. Boyne M. T. Podevels A. M. Klimowicz A. K. Handelsman J. Kelleher N. L. (2006). Hydroxymalonyl-acyl carrier protein (ACP) and aminomalonyl-ACP are two additional type I polyketide synthase extender units. *Proc. Natl. Acad. Sci. U.S.A.* 103 14349–14354. 10.1073/pnas.0603748103 16983083 PMC1599966

[B11] Covington C. Polavarapu P. (2013). Similarity in dissymmetry factor spectra: a quantitative measure of comparison between experimental and predicted vibrational circular dichroism. *J. Phys. Chem. A* 117 3377–3386. 10.1021/jp401079s 23534955

[B12] Covington C. Polavarapu P. (2014). CDSpecTech: Computer Programs for Calculating Similarity Measures of Comparison between Experimental and Calculated Dissymmetry Factors and Differentials. Available at: https://sites.google.com/site/cdspectech1/

[B13] Daum M. Peintner I. Linnenbrink A. Frerich A. Weber M. Paululat T. (2009). Organisation of the biosynthetic gene cluster and tailoring enzymes in the biosynthesis of the tetracyclic quinone glycoside antibiotic polyketomycin. *Chembiochem* 10 1073–1083. 10.1002/cbic.200800823 19266534

[B14] Demain A. L. Fang A. (2000). The natural functions of secondary metabolites. *Adv. Biochem. Eng. Biotechnol.* 69 1–39. 11036689 10.1007/3-540-44964-7_1

[B15] Diarra M. S. Lavoie M. C. Jacques M. Darwish I. Dolence E. K. Dolence J. A. (1996). Species selectivity of new siderophore-drug conjugates that use specific iron uptake for entry into bacteria. *Antimicrob. Agents Chemother.* 40 2610–2617. 8913474 10.1128/aac.40.11.2610 PMC163585

[B16] Donadio S. Sosio M. Stegmann E. Weber T. Wohlleben W. (2005). Comparative analysis and insights into the evolution of gene clusters for glycopeptide antibiotic biosynthesis. *Mol. Genet. Genomics* 274 40–50. 10.1007/s00438-005-1156-3 16007453

[B17] Doroghazi J. R. Albright J. C. Goering A. W. Ju K.-S. Haines R. R. Tchalukov K. A. (2014). A roadmap for natural product discovery based on large-scale genomics and metabolomics. *Nat. Chem. Biol.* 10 963–968. 10.1038/nchembio.1659 25262415 PMC4201863

[B18] Dorrestein P. C. Van Lanen S. G. Li W. Zhao C. Deng Z. Shen B. (2006). The bifunctional glyceryl transferase/phosphatase OzmB belonging to the HAD superfamily that diverts 1,3-bisphosphoglycerate into polyketide biosynthesis. *J. Am. Chem. Soc.* 128 10386–10387. 10.1021/ja0639362 16895402

[B19] Doyle T. W. Balitz D. M. Grulich R. E. Nettleton D. E. Gould S. J. Tann C. (1981). Structure determination of lavendamycin- a new antitumor antibiotic from *Streptomyces lavendulae*. *Tetrahedron Lett.* 22 4595–4598. 10.1016/S0040-4039(01)82990-7

[B20] Duchaud E. Rusniok C. Frangeul L. Buchrieser C. Givaudan A. Taourit S. (2003). The genome sequence of the entomopathogenic bacterium *Photorhabdus luminescens*. *Nat. Biotechnol.* 21 1307–1313. 10.1038/nbt886 14528314

[B21] Egan S. Wiener P. Kallifidas D. Wellington E. M. (2001). Phylogeny of *Streptomyces* species and evidence for horizontal transfer of entire and partial antibiotic gene clusters. *Antonie Van Leeuwenhoek* 79 127–133. 10.1023/A:1010296220929 11519998

[B22] Firn R. D. Jones C. G. (2000). The evolution of secondary metabolism - a unifying model. *Mol. Microbiol.* 37 989–994. 10.1046/j.1365-2958.2000.02098.x 10972818

[B23] Fischbach M. A. Walsh C. T. Clardy J. (2008). The evolution of gene collectives: how natural selection drives chemical innovation. *Proc. Natl. Acad. Sci. U.S.A.* 105 4601–4608. 10.1073/pnas.0709132105 18216259 PMC2290807

[B24] Flett F. Mersinias V. Smith C. P. (1997). High efficiency intergeneric conjugal transfer of plasmid DNA from *Escherichia coli* to methyl DNA-restricting streptomycetes. *FEMS Microbiol. Lett.* 155 223–229. 10.1016/S0378-1097(97)00392-3 9351205

[B25] Floss H. Yu T. Arakawa K. (2011). The biosynthesis of 3-amino-5-hydroxybenzoic acid (AHBA), the precursor of mC7N units in ansamycin and mitomycin antibiotics: a review. *J. Antibiot. (Tokyo)* 64 35–44. 10.1038/ja.2010.139 21081954

[B26] Frisch M. J. Trucks G. W. Schlegel H. B. Scuseria G. E. Robb M. A. Cheeseman J. R. (2013). *Gaussian 09 R-D.01*. Wallingford, CT: Gaussian Inc.

[B27] Gessner A. Heitzler T. Zhang S. Klaus C. Murillo R. Zhao H. (2015). Changing biosynthetic profiles by expressing *bldA* in *Streptomyces* strains. *Chembiochem* 16 2244–2252. 10.1002/cbic.201500297 26255983

[B28] Gomez-Escribano J. P. Bibb M. J. (2014). Heterologous expression of natural product biosynthetic gene clusters in *Streptomyces coelicolor*: from genome mining to manipulation of biosynthetic pathways. *J. Ind. Microbiol. Biotechnol.* 41 425–431. 10.1007/s10295-013-1348-5 24096958

[B29] Grüning B. A. Erxleben A. Hähnlein A. Günther S. (2013). Draft genome sequence of *Streptomyces viridochromogenes* strain Tü57, producer of avilamycin. *Genome Announc.* 1:e00384-13 10.1128/genomeA.00384-13 PMC3707599 23788550

[B30] Hütter R. (1962). Zur Systematik der Actinomyceten. *Arch. Mikrobiol.* 43 23–49. 10.1007/BF00408394 14449748

[B31] Ikeda H. Ishikawa J. Hanamoto A. Shinose M. Kikuchi H. Shiba T. (2003). Complete genome sequence and comparative analysis of the industrial microorganism *Streptomyces avermitilis*. *Nat. Biotechnol.* 21 526–531. 10.1038/nbt820 12692562

[B32] Intra B. Matsumoto A. Inahashi Y. Omura S. Takahashi Y. Panbangred W. (2013). *Actinokineospora bangkokensis* sp. nov., isolated from rhizospheric soil. *Int. J. Syst. Evol. Microbiol.* 63 2655–2660. 10.1099/ijs.0.047928-0 23291892

[B33] Kawamukai M. (2002). Biosynthesis, bioproduction and novel roles of ubiquinone. *J. Biosci. Bioeng.* 94 511–517. 10.1016/S1389-1723(02)80188-8 16233343

[B34] Klingeman D. M. Utturkar S. Lu T.-Y. S. Schadt C. W. Pelletier D. A. Brown S. D. (2015). Draft genome sequences of four *Streptomyces* isolates from the *Populus trichocarpa* root endosphere and rhizosphere. *Genome Announc.* 3:e1344-15 10.1128/genomeA.01344-15 PMC4972787 26564053

[B35] Kobayashi J. Madono T. Shigemori H. (1995). Nakijiquinones C and D, new sesquiterpenoid quinones with a hydroxy amino acid residue from a marine sponge inhibiting c-erbB-2 kinase. *Tetrahedron* 51 10867–10874. 10.1016/0040-4020(95)00661-Q

[B36] Kong F. Zhao N. Siegel M. M. Janota K. Ashcroft J. S. Koehn F. E. (1998). Saccharomicins, novel heptadecaglycoside antibiotics effective against multidrug-resistant bacteria. *J. Am. Chem. Soc.* 120 13301–13311. 10.1021/ja981641l

[B37] Kusebauch B. Busch B. Scherlach K. Roth M. Hertweck C. (2010). Functionally distinct modules operate two consecutive α,β→β,γ double-bond shifts in the rhizoxin polyketide assembly line. *Angew. Chemie* 122 1502–1506. 10.1002/ange.200905467 20033973

[B38] Lin P. Li S. Wang S. Yang Y. Shi J. (2006). A nitrogen-containing 3-alkyl-1,4-benzoquinone and a gomphilactone derivative from *Embelia ribes*. *J. Nat. Prod.* 69 1629–1632. 10.1021/np060284m 17125236

[B39] Llewellyn N. M. Spencer J. B. (2006). Biosynthesis of 2-deoxystreptamine-containing aminoglycoside antibiotics. *Nat. Prod. Rep.* 23 864–874. 10.1039/b604709m 17119636

[B40] Lohr F. Jenniches I. Frizler M. Meehan M. J. Sylvester M. Schmitz A. (2013). α,β → β,γ double bond migration in corallopyronin A biosynthesis. *Chem. Sci.* 4:4175 10.1039/c3sc51854j

[B41] MacNeil D. J. Gewain K. M. Ruby C. L. Dezeny G. Gibbons P. H. MacNeil T. (1992). Analysis of *Streptomyces avermitilis* genes required for avermectin biosynthesis utilizing a novel integration vector. *Gene* 111 61–68. 10.1016/0378-1119(92)90603-M 1547955

[B42] Makitrynskyy R. Ostash B. Tsypik O. Rebets Y. Doud E. Meredith T. (2013). Pleiotropic regulatory genes *bldA, adpA* and *absB* are implicated in production of phosphoglycolipid antibiotic moenomycin. *Open Biol.* 3:130121 10.1098/rsob.130121 PMC3814723 24153004

[B43] Matsunaga E. Higuchi Y. Mori K. Tashiro K. Kuhara S. Takegawa K. (2015). Draft genome sequence of *Streptomyces* sp. JHA19, a strain that possesses β-D-galactofuranosidase activity. *Genome Announc.* 3:e1171-15 10.1128/genomeA.01171-15 PMC4599098 26450739

[B44] Metsä-Ketelä M. Halo L. Munukka E. Hakala J. Mäntsälä P. Ylihonko K. (2002). Molecular evolution of aromatic polyketides and comparative sequence analysis of polyketide ketosynthase and 16S ribosomal DNA genes from various streptomyces species. *Appl. Environ. Microbiol.* 68 4472–4479. 10.1128/AEM.68.9.4472-4479.2002 12200302 PMC124067

[B45] Moldenhauer J. Götz D. C. G. Albert C. R. Bischof S. K. Schneider K. Süssmuth R. D. (2010). The final steps of bacillaene biosynthesis in *Bacillus amyloliquefaciens* FZB42: direct evidence for β,γ dehydration by a trans-acyltransferase polyketide synthase. *Angew. Chem. Int. Ed. Engl.* 49 1465–1467. 10.1002/anie.200905468 20087918

[B46] Möllmann U. Heinisch L. Bauernfeind A. Köhler T. Ankel-Fuchs D. (2009). Siderophores as drug delivery agents: application of the “Trojan Horse” strategy. *Biometals* 22 615–624. 10.1007/s10534-009-9219-2 19214755

[B47] Oppolzer W. Prelog V. (1973). The constitution and configuration of rifamycins B, O, S and SV. *Helv. Chim. Acta* 56 2287–2314. 10.1002/hlca.19730560717 4761272

[B48] Oshima K. Hattori M. Shimizu H. Fukuda K. Nemoto M. Inagaki K. (2015). Draft genome sequence of *Streptomyces incarnatus* NRRL8089, which produces the nucleoside antibiotic sinefungin. *Genome Announc.* 3:e00715-15 10.1128/genomeA.00715-15 PMC4498112 26159526

[B49] Paululat T. Zeeck A. Gutterer J. M. Fiedler H. P. (1999). Biosynthesis of polyketomycin produced by *Streptomyces diastatochromogenes* Tü6028. *J. Antibiot. (Tokyo)* 52 96–101. 10.7164/antibiotics.52.96 10344562

[B50] Pelludat C. Rakin A. Jacobi C. A. Schubert S. Heesemann J. (1998). The yersiniabactin biosynthetic gene cluster of *Yersinia enterocolitica*: organization and siderophore-dependent regulation. *J. Bacteriol.* 180 538–546. 9457855 10.1128/jb.180.3.538-546.1998 PMC106919

[B51] Rao K. V. Cullen W. P. (1959–1960). Streptonigrin, an antitumor substance. I. Isolation and characterization. *Antibiot. Annu.* 7 950–953. 14436228

[B52] Rauchová H. Vrbackı M. Bergamini C. Fato R. Lenaz G. Houstek J. (2006). Inhibition of glycerophosphate-dependent H_2_O_2_ generation in brown fat mitochondria by idebenone. *Biochem. Biophys. Res. Commun.* 339 362–366. 10.1016/j.bbrc.2005.11.035 16300743

[B53] Rinehart K. L. Shield L. S. (1976). Chemistry of the ansamycin antibiotics. *Fortschr. Chem. Org. Naturst.* 33 231–307. 11155 10.1007/978-3-7091-3262-3_3

[B54] Rückert C. Szczepanowski R. Albersmeier A. Goesmann A. Iftime D. Musiol E. M. (2013). Complete genome sequence of the kirromycin producer *Streptomyces collinus* Tü 365 consisting of a linear chromosome and two linear plasmids. *J. Biotechnol.* 168 739–740. 10.1016/j.jbiotec.2013.10.004 24140291

[B55] Schroeckh V. Scherlach K. Nützmann H.-W. Shelest E. Schmidt-Heck W. Schuemann J. (2009). Intimate bacterial-fungal interaction triggers biosynthesis of archetypal polyketides in *Aspergillus nidulans*. *Proc. Natl. Acad. Sci. U.S.A.* 106 14558–14563. 10.1073/pnas.0901870106 19666480 PMC2732885

[B56] Schulz D. Beese P. Ohlendorf B. Erhard A. Zinecker H. Dorador C. (2011). Abenquines A–D: aminoquinone derivatives produced by *Streptomyces* sp. strain DB634. *J. Antibiot. (Tokyo)* 64 763–768. 10.1038/ja.2011.87 21952099

[B57] Schwarzer D. Finking R. Marahiel M. A. (2003). Nonribosomal peptides: from genes to products. *Nat. Prod. Rep.* 20 275–287. 10.1039/b111145k 12828367

[B58] Schwyn B. Neilands J. B. (1987). Universal chemical assay for the detection and determination of siderophores. *Anal. Biochem.* 160 47–56. 10.1016/0003-2697(87)90612-9 2952030

[B59] Shigemori H. Madono T. Sasaki T. Mikami Y. Kobayashi J. (1994). Nakijiquinones A and B, new antifungal sesquiterpenoid quinones with an amino acid residue from an Okinawan marine sponge. *Tetrahedron* 50 8347–8354. 10.1016/S0040-4020(01)85557-5

[B60] Sievers F. Wilm A. Dineen D. Gibson T. J. Karplus K. Li W. (2011). Fast, scalable generation of high-quality protein multiple sequence alignments using Clustal Omega. *Mol. Syst. Biol.* 7:539 10.1038/msb.2011.75 PMC3261699 21988835

[B61] Spiteller P. Arnold N. Spiteller M. Steglich W. (2003). Lilacinone, a red aminobenzoquinone pigment from *Lactarius lilacinus*. *J. Nat. Prod.* 66 1402–1403. 10.1021/np0303052 14575448

[B62] Staunton J. Weissman K. J. (2001). Polyketide biosynthesis: a millennium review. *Nat. Prod. Rep.* 18 380–416. 10.1039/a909079g 11548049

[B63] Sugiyama Y. Fujita T. Matsumoto M. Okamoto K. Imada I. (1985). Effects of idebenone (CV-2619) and its metabolites on respiratory activity and lipid peroxidation in brain mitochondria from rats and dogs. *J. Pharmacobiodyn.* 8 1006–1017. 10.1248/bpb1978.8.1006 2871147

[B64] Sun Y. Hong H. Gillies F. Spencer J. B. Leadlay P. F. (2008). Glyceryl-S-acyl carrier protein as an intermediate in the biosynthesis of tetronate antibiotics. *Chembiochem* 9 150–156. 10.1002/cbic.200700492 18046685

[B65] Suno M. Nagaoka A. (1984). Inhibition of lipid peroxidation by a novel compound (CV-2619) in brain mitochondria and mode of action of the inhibition. *Biochem. Biophys. Res. Commun.* 125 1046–1052. 10.1016/0006-291X(84)91389-5 6517932

[B66] Taft F. Brünjes M. Knobloch T. Floss H. G. Kirschning A. (2009). Timing of the Δ_1_0,12-Δ_1_1,13 double bond migration during ansamitocin biosynthesis in *Actinosynnema pretiosum*. *J. Am. Chem. Soc.* 131 3812–3813. 10.1021/ja8088923 19292483

[B67] Takahashi Y. Kubota T. Ito J. Mikami Y. Fromont J. Kobayashi J. (2008). Nakijiquinones G-I, new sesquiterpenoid quinones from marine sponge. *Bioorg. Med. Chem.* 16 7561–7564. 10.1016/j.bmc.2008.07.028 18676149

[B68] Takahashi Y. Kubota T. Kobayashi J. (2009). Nakijiquinones E and F, new dimeric sesquiterpenoid quinones from marine sponge. *Bioorg. Med. Chem.* 17 2185–2188. 10.1016/j.bmc.2008.10.080 19017563

[B69] Udwary D. W. Zeigler L. Asolkar R. N. Singan V. Lapidus A. Fenical W. (2007). Genome sequencing reveals complex secondary metabolome in the marine actinomycete *Salinispora tropica*. *Proc. Natl. Acad. Sci. U.S.A.* 104 10376–10381. 10.1073/pnas.0700962104 17563368 PMC1965521

[B70] Vaishnav P. Demain A. L. (2011). Unexpected applications of secondary metabolites. *Biotechnol. Adv.* 29 223–229. 10.1016/j.biotechadv.2010.11.006 21130862

[B71] Waksman S. A. Gregory F. J. (1954). Actinomycin. II. Classification of organism producing different forms of actinomycin. *Antibiot. Chemother. (Northfield)* 4 1050–1056. 24543239

[B72] Wang X.-J. Yan Y.-J. Zhang B. An J. Wang J.-J. Tian J. (2010). Genome sequence of the milbemycin-producing bacterium *Streptomyces bingchenggensis*. *J. Bacteriol.* 192 4526–4527. 10.1128/JB.00596-10 20581206 PMC2937363

[B73] Webb J. S. Cosulich D. B. Mowat J. H. Patrick J. B. Broschard R. W. Meyer W. E. (1962). The structures of mitomycins A, B and C and porfiromycin–part I. *J. Am. Chem. Soc.* 84 3185–3187. 10.1021/ja00875a032

[B74] Weber T. Blin K. Duddela S. Krug D. Kim H. U. Bruccoleri R. (2015a). antiSMASH 3.0 - a comprehensive resource for the genome mining of biosynthetic gene clusters. *Nucleic Acids Res.* 43 W237–W243. 10.1093/nar/gkv437 25948579 PMC4489286

[B75] Weber T. Charusanti P. Musiol-Kroll E. M. Jiang X. Tong Y. Kim H. U. (2015b). Metabolic engineering of antibiotic factories: new tools for antibiotic production in actinomycetes. *Trends Biotechnol.* 33 15–26. 10.1016/j.tibtech.2014.10.009 25497361

[B76] Weiss R. F. (1970). The solubility of nitrogen, oxygen and argon in water and seawater. *Deep Sea Res. Oceanogr. Abstr.* 17 721–735. 10.1016/0011-7471(70)90037-9

[B77] White R. J. Martinelli E. Gallo G. G. Lancini G. Beynon P. (1973). Rifamycin biosynthesis studied with ^13^C enriched precursors and carbon magnetic resonance. *Nature* 243 273–277. 10.1038/243273a0 4743213

