# Supplementary material for: Wide Distribution of Foxicin Biosynthetic Gene Clusters in Streptomyces Strains – An Unusual Secondary Metabolite with Various Properties
Source: Front Microbiol. 2017 Feb 21;8:221. doi: 10.3389/fmicb.2017.00221 (PMC5318452; doi:10.3389/fmicb.2017.00221)
Supplement: Supplementary file 1 [file Data_Sheet_1.docx]

***Supplementary Material***

**Wide distribution of foxicin biosynthetic gene clusters in *Streptomyces* strains – an unusual secondary metabolite with various properties**

**Anja Greule^1^, Marija Marolt^2^, Denise Deubel^1^, Iris Peintner^1^, Songya Zhang^1^, Claudia Jessen-Trefzer^1^, Christian De Ford^1,3^, Sabrina Burschel^4^, Shu-Ming Li^5^, Thorsten Friedrich^4^, Irmgard Merfort^1^, Steffen Lüdeke^2^, Philippe Bisel^2^, Michael Müller^2^, Thomas Paululat^6^, Andreas Bechthold^1*^**

^1^Department of Pharmaceutical Biology and Biotechnology, Albert-Ludwigs-University of Freiburg, Freiburg im Breisgau, Germany;

^2^Department of Pharmaceutical and Medical Chemistry, Albert-Ludwigs-University of Freiburg, Freiburg im Breisgau, Germany;

^3^Spemann Graduate School of Biology and Medicine (SGBM), Albert-Ludwigs-University of Freiburg, Freiburg im Breisgau, Germany;

^4^Institute of Biochemistry, Albert-Ludwigs-University of Freiburg, Freiburg im Breisgau, Germany;

^5^Department of Pharmaceutical Biology, Philipps-University Marburg, Marburg, Germany;

^6^Department of Chemistry and Biology, University of Siegen, Siegen, Germany.

***Correspondence:**

Andreas Bechthold

[andreas.bechthold@pharmazie.uni-freiburg.de](mailto:andreas.bechthold@pharmazie.uni-freiburg.de)

**1. Supplementary Tables**

**Table S1. Physico-chemical properties of foxicin A**

| Appearance | Red powder |
| --- | --- |
| CI-MS *m/z* | 346 (M, C_18_H_22_N_2_O_5_), 345 (M-H)^-^, 347 (M+H)^+^ |
| HR-EIMS *m/z*  Calculated:  Found: | 346,1529 (as C_18_H_22_N_2_O_5_)  346,1532 |
| UV $\text{E}_{\text{1cm}}^{\text{1\%}}$  ($\text{λ}_{\text{202nm}}^{\text{MeOH}}$) $\text{λ}_{\text{max}}^{\text{MeOH}}$  [nm] | 998  202, 227 (sh, 241), 315, 479 |
| NMR | Data are shown in Table S2+S3 |
| Soluble in | MeOH, ACN, CHCl_3_ and DMSO |
| Unsoluble in | water |

**Table S2. NMR data of foxicin A (600/150/60MHz, CDCl_3_, 25 °C)**

| **Pos.** | **δ_C_** | **δ_N_^a^** | **δ_H ;_**  **mult (J in Hz)** | **COSY** | **H2BC^b^** | **^1^H,^13^C-HMBC &**  **^1^H,^15^N-HMBC^b^** |
| --- | --- | --- | --- | --- | --- | --- |
| **1** | 178.6 |  |  |  |  | 5-H, (1‘-NH), 1‘‘-NH |
| **2** | 116.0 |  |  |  |  | (5-H) |
| **3** | 145.5 |  |  |  |  | 5-H, 1‘-NH |
| **3-OH** |  |  | 13.00 br s |  |  |  |
| **4** | 182.3 |  |  |  |  | (1‘‘-H) |
| **5** | 111.8 |  | 7.46 s |  | ^1^J | 1‘‘-NH, ^1^J |
| **6** | 137.2 |  |  |  |  | 5-H |
| **1ʹ-NH** |  | 122.6 | 7.96 s |  |  | 3‘-H_3_, ^1^J |
| **2ʹ** | 171.6 |  |  |  |  | (1‘-NH), 3‘-H_3_ |
| **3ʹ** | 23.6 |  | 2.31 s |  |  | ^1^J |
| **1ʹʹ-NH** |  | 116.6 | 8.56 s |  |  | ^1^J |
| **2ʹʹ** | 167.5 |  |  |  |  | 1‘‘-NH, 3‘‘-CH_3_, 4‘‘-H |
| **3ʹʹ** | 128.2 |  |  |  |  | 3‘‘-CH_3_, 5‘‘-H |
| **3ʹʹ-CH_3_** | 12.5 |  | 1.96 d (0.8) |  | 4‘‘-H | 4‘‘-H, ^1^J |
| **4ʹʹ** | 144.2 |  | 6.30 dd  (9.6, 0.8) | 3‘‘-CH_3_, 5‘‘-H | ^1^J, 3‘‘-CH_3_, 5‘‘-H, | 3‘‘-CH_3_, 5‘‘-H, 5‘‘-CH_3_, 6‘‘-H |
| **5ʹʹ** | 32.7 |  | 3.40 m | 4‘‘-H, 5‘‘-CH_3_, 6‘‘-H | ^1^J, 4‘‘-H, 5‘‘-CH_3_, 6‘‘-H | 3‘‘-CH_3_, 4‘‘-H, 5‘‘-H, 5‘‘-CH_3_, 7‘‘-CH_3_, 8‘‘-H_3_, ^1^J |
| **5ʹʹ-CH_3_** | 20.8 |  | 1.08 d (6.8) | 5‘‘-H | ^1^J, 5‘‘-H | 3‘‘-CH_3_, 4‘‘-H, 5‘‘-H, 6‘‘-H, ^1^J |
| **6ʹʹ** | 126.7 |  | 5.01 br d (8.8) | 5‘‘-H, 7‘‘-CH_3_, 8‘‘-H_3_ | ^1^J, 5‘‘-H, 7‘‘-CH_3,_ 8‘‘-H_3_ | 4‘‘-H, 5‘‘-H, 3‘‘-CH_3_, 5‘‘-CH_3_, 7‘‘-CH_3_, 8‘‘-H_3_, |
| **7ʹʹ** | 132.3 |  |  |  |  | 4‘‘-H |
| **7ʹʹ-CH_3_** | 18.1 |  | 1.64 d (0.8) | 6‘‘-H | ^1^J, 6‘‘-H | 6‘‘-H, 8‘‘-H_3_ |
| **8ʹʹ** | 25.7 |  | 1.70 s | 6‘‘-H | ^1^J, 6‘‘-H,  (7‘‘-CH_3_) | 6‘‘-H, 7‘‘-CH_3_ |

^a^ weak signals in brackets; abbreviations: s=singlet, d=doublet, dd=doublet of doublets, m=multiplet, br=broad.

**Table S3.** **NMR data of foxicin A (600/150MHz, DMSO-d_6_, 35 °C)**

| **Pos.** | **δ_C_** | **δ_H ;_**  **mult (J in Hz)** | **COSY** | **^1^H,^13^C-HMBC^a^** |
| --- | --- | --- | --- | --- |
| **1** | 177.8 |  |  | 5-H, 1’’-NH |
| **2** | 116.0 |  |  | (5-H) |
| **3** | 151.7 br |  |  | 5-H |
| **3-OH** |  | missing |  |  |
| **4** | 183.4 |  |  | (1’’-NH) |
| **5** | 109.4 | 7.18 s |  | 1‘‘-NH |
| **6** | 139.2 |  |  | 5-H |
| **1ʹ-NH** |  | 9.15 br s |  |  |
| **2ʹ** | 169.3 |  |  | 3‘-H_3_ |
| **3ʹ** | 22.4 | 2.00 s |  |  |
| **1ʹʹ-NH** |  | 8.99 br s |  |  |
| **2ʹʹ** | 167.6 |  |  | 1‘‘-NH, 3‘‘-CH_3_, 4‘‘-H |
| **3ʹʹ** | 128.0 |  |  | 3‘‘-CH_3_, 5‘‘-H |
| **3ʹʹ-CH_3_** | 12.2 | 1.90 d (1.2) |  | ^1^J, 4‘‘-H |
| **4ʹʹ** | 143.4 | 6.33 dd (9.2, 1.2) | 3‘‘-CH_3_, 5‘‘-H | 3‘‘-CH_3_, 5‘‘-H, 5‘‘-CH_3_, (6‘‘-H), 7‘‘-CH_3_, 8‘‘-H_3_ |
| **5ʹʹ** | 32.1 | 3.42 m | 5‘‘-CH_3_, 6‘‘-H | 3‘‘-CH_3_, 4‘‘-H, 5‘‘-CH_3_, 6‘‘-H, (7‘‘-CH_3_, 8‘‘-H_3_) |
| **5ʹʹ-CH_3_** | 20.6 | 1.05 d (6.8) | 5‘‘-H | 3‘‘-H_3_, 4‘‘-H, 5‘‘-H, 6‘‘-H, (8‘‘-H_3_) |
| **6ʹʹ** | 127.2 | 5.07 dm (8.8,m) | 5‘‘-H, 7‘‘-CH_3_, 8‘‘-H_3_ | (3‘‘-CH_3_), 4‘‘-H, 5‘‘-H, 5‘‘-CH_3_, 7‘‘-CH_3_, 8‘‘-H_3_ |
| **7ʹʹ** | 131.1 |  |  | 5‘‘-H, 5‘‘-H_3_, 8‘‘-H_3_ |
| **7ʹʹ-CH_3_** | 17.8 | 1.63 d (1.2) | 6‘‘-CH_3_ | 6‘‘-H, ^1^J, 8‘‘-H_3_ |
| **8ʹʹ** | 25.4 | 1.66 d (0.8) | 6‘‘-H | 6‘‘-H, ^1^J, 7‘‘-CH_3_ |

^a^ weak signals in brackets; abbreviations: s=singlet, d=doublet, dd=doublet of doublets, m=multiplet, br=broad.

**Table S4: Similar fox biosynthetetic gene cluster in other Streptomyces strains**

| **Strain** | **GenBank assembly accession** | **Taxonomy ID** | **Known product** | **Percent Identity to…** | | | | |
| --- | --- | --- | --- | --- | --- | --- | --- | --- |
|  |  |  |  | ***foxBI*** | ***foxBII*** | ***foxBIII*** | ***foxEI*** | ***foxEII*** |
| *Streptomyces aureus*  NRRL B-1941 | GCA_000725495.1 |  | (Phosphonic acid)  (Doroghazi et al., 2014)^,^(Ju et al., 2015)) | 82.49 | 78.23 | 75.65 | 83.82 | 82.44 |
| *Streptomyces avermitilis*  MA4680 | GCA_000009765.2 | 227882 | Avermectin (Burg et al., 1979) | 86.16 | 82.08 | 82.56 | 87.06 | 85.33 |
| *Streptomyces bicolor*  NRRL B-5348 | GCA_000719285.1 |  | (Phosphonic acid)  (Doroghazi et al., 2014)^,^(Ju et al., 2015) | 81.87 | 79.19 | 77.42 | 85.05 | 85.77 |
| *Streptomyces bingchenggensis* BCW-1 | GCA_000092385.1 | 749414 | Milbemycin(Wang et al., 2009), Bingchamide(Xiang et al., 2009), Nanchangmycin(Zhang et al., 2013) | 77.34 | 75.91 | 73.25 | 79.60 | 79.38 |
| *Streptomyces bungoensis*  DSM 41781 | GCA_001514215.1 | 285568 |  | 81.58 | 78.76 | 73.15 | 83.64 | 82.69 |
| *Streptomyces caeruleatus*  NRRL B-24802 | GCA_001514235.1 | 661399 | dark blue pigments, Melanin, antibacterial compound (Zhu et al., 2011) | 80.43 | 78.91 | 75.21 | 84.96 | 84.62 |
| *Streptomyces cellostaticus*  DSM 40189 | GCA_001513965.1 | 67285 | [Cellostatin](https://en.wikipedia.org/w/index.php?title=Cellostatin&action=edit&redlink=1) (Hamada, 1958; Hamada and Sato, 1958) | 82.05 | 79.44 | 71.77 | 84.34 | 87.31 |
| *Streptomyces collinus*  Tü365 | GCA_000444875.1 | 1214242 | Kirromycin (Weber et al., 2008; Wolf and Zähner, 1972), Streptocollin (Iftime et al., 2015), Isorenieratene, Desferrioxamin E, Deoxydehydrochorismic acid, Pentalenolacton, Hopene/squalene (Iftime et al., 2016) | 81.18 | 78.34 | 73.01 | 84.70 | 84.62 |
| *Streptomyces curacoi*  DSM 40107 | GCA_001513975.1 | 146536 | Curamycin  (Galmarini and Deulofeu, 1961; Gros et al., 1968) | 80.55 | 80.00 | 76.09 | 85.22 | 85.13 |
| *Streptomyces incarnatus*  NRRL 8089 | GCA_001027185.1 | 665007 | Sinefungin  (Oshima et al., 2015)^,^(Florent et al., 1967) | 83.39 | 80.89 | 81.44 | 85.75 | 0.0 |
| *Streptomyces mangrovisoli*  DSM 100438 | GCA_000974985.1 | 1428628 | Pyrrolo[1,2-a]pyrazine-1,4-dione, hexahydro-  (Ser et al., 2015) | 80.16 | 77.17 | - | - | - |
| *Streptomyces olivochromogenes* DSM 40451 | GCA_001514115.1 | 1963 |  | 84.71 | 81.92 | 78.40 | 85.48 | 83.21 |
| *Streptomyces puniciscabiei*  NRRL B-24456 | GCA_001419685.1 | 164348 |  | 83.28 | 80.14 | 79.04 | 85.40 | 0.0 |
| *Streptomyces regalis* NRRL 3151 | GCA_001509475.1 | 68262 |  | 81.82 | 79.40 | 76.27 | 84.61 | 85.90 |
| *Streptomyces viridochromogenes* NRRL_3413 | GCA_001270495.1 |  | Fosfomycin  (Hendlin et al., 1969; White and Demain, 1976) | 80.84 | 79.02 | - | - | - |
| *Streptomyces viridochromogenes* NRRL 3414 | GCA_001047325.1 |  | Fosfomycin  (Hendlin et al., 1969; White and Demain, 1976) | 80.84 | 79.04 | 76.51 | 85.40 | 83.21 |
| *Streptomyces viridochromogenes* NRRL 3416 | GCA_001270485.1 |  | Fosfomycin  (Hendlin et al., 1969; White and Demain, 1976) | 80.84 | 79.02 | 76.60 | - | - |
| *Streptomyces viridochromogenes* Tü57 | GCA_000332625.1 | 1160705 | Avilamycin (Buzzetti et al., 1968; Gaisser et al., 1997) | 80.33 | 79.70 | 76.85 | 85.40 | 86.28 |
| *Streptomyces* sp. 142MFCol3.1 | GCA_000424945.1 | 1172179 |  | 96.75 | 94.79 | 93.40 | 95.69 | 96.41 |
| *Streptomyces* sp. JHA19 | GCA_001417695.1 | 1577588 |  | 81.04 | 76.96 | 74.78 | 84.48 | 82.18 |
| *Streptomyces* sp. OK006 | GCA_001298565.1 | 1592326 |  | 84.88 | 81.60 | 77.70 | 85.74 | 82.95 |

**Table S5. Inhibition of *in vitro* respiration of *E. coli* membranes by foxicin A**

| **Foxicin A concentration [µM]** | **O_2_ consumption [µM/min]** | **Activity** | | **Percentage %** |
| --- | --- | --- | --- | --- |
|  |  | **Units [µmol/min]** | **Units / mg** |  |
| **0** | 140.24 | 0.2805 | - | 100 |
| **50** | 110.03 | 0.2201 | 6.36 | 78.5 |
| **100** | 99.58 | 0.1992 | 2.88 | 71.0 |
| **250** | 88.10 | 0.1762 | 1.02 | 62.8 |
| **500** | 43.09 | 0.0862 | 0.25 | 30.7 |

**Table S6. Agar plate diffusion assay with foxicin A**

| **Bacteria strain** | **Substance [µg/disc]** | **Inhibition zone [cm]** | **Activity** |
| --- | --- | --- | --- |
| ***Actinokineospora bangkokensis*** | Foxicin A [100] | - | - |
|  | Apramycin [25] | - | - |
|  | Methanol | - | - |
| ***S. viridochromogenes* Tü57** | **Foxicin A [100]** | **0.7** | **+** |
|  | Polyketomycin [100] | 1.4 | + |
|  | Apramycin [25] | 1.7 | + |
|  | Methanol | - | - |
| ***Saccharothrix espanaensis*** | **Foxicin A [100]** | **0.65** | **+** |
|  | Apramycin [25] | 2 | + |
|  | Methanol | - | - |
| ***B. subtilis***  **COHN ATCC6051** | Foxicin A [100] | - | - |
|  | Polyketomycin [100] | 2.1 | + |
|  | Apramycin [25] | 2.3 | + |
|  | Methanol | - | - |
| ***E. coli* XL1-Blue** | Foxicin A [100] | - | - |
|  | Apramycin [25] | 2.0 | + |
|  | Methanol | - | - |
| ***Mycobacterium smagmatis*** | Foxicin [100] | - | - |
|  | Methanol | - | - |
| ***Synechococcus* sp. PCC7002** | **Foxicin A [100]** | **0.7** | **+** |
|  | Chloramphenicol [300] | 1.1 | + |
|  | Methanol | - | - |
| ***Synechocystis* sp. PCC6803** | **Foxicin A [100]** | **0.65** | **+** |
|  | Chloramphenicol [300] | 1.5 | + |
|  | Methanol | - | - |
| ***Candida parapsilosis*** | Foxicin A [100] | - | - |
|  | Methanol | - | - |
| ***Fusarium* *verticilloides*** | Foxicin A [100] | - | - |
|  | Methanol [20] | - | - |

+ antibiotic active; - antibiotic not active; **red**: foxicin is antibacterial active

**2. Supplementary Figures**

**Figure S1: UV/vis spectra and mass spectra of polyketomycin and foxicin derivates**

Top: UV/vis spectra, below: negative mass spectra, from left to right; polyketomycin, foxicin A, foxicin B, foxicin C and foxicin D.


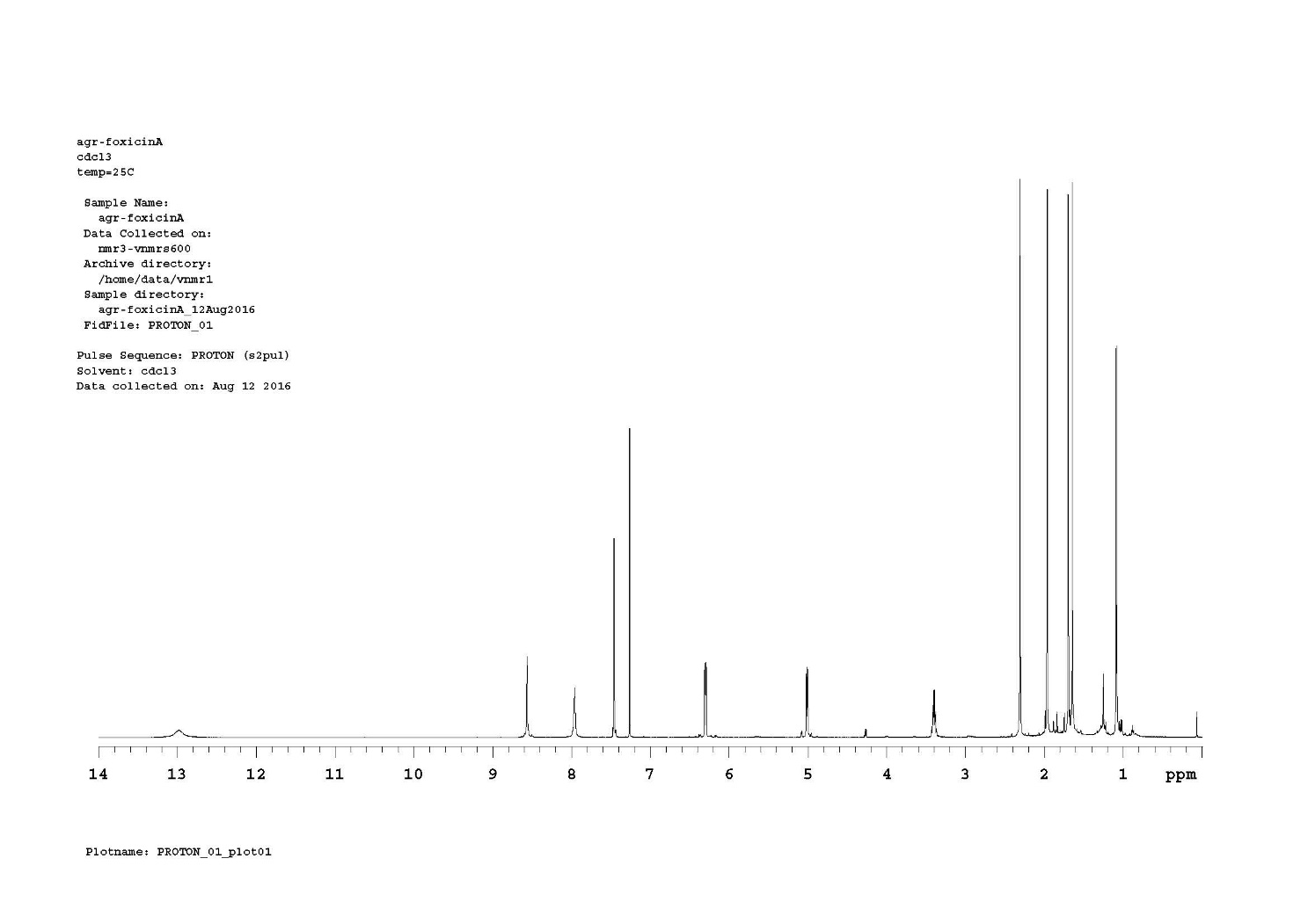


**Figure S2: ^1^H NMR of foxicin A (600MHz, CDCl_3_, 25 °C)**


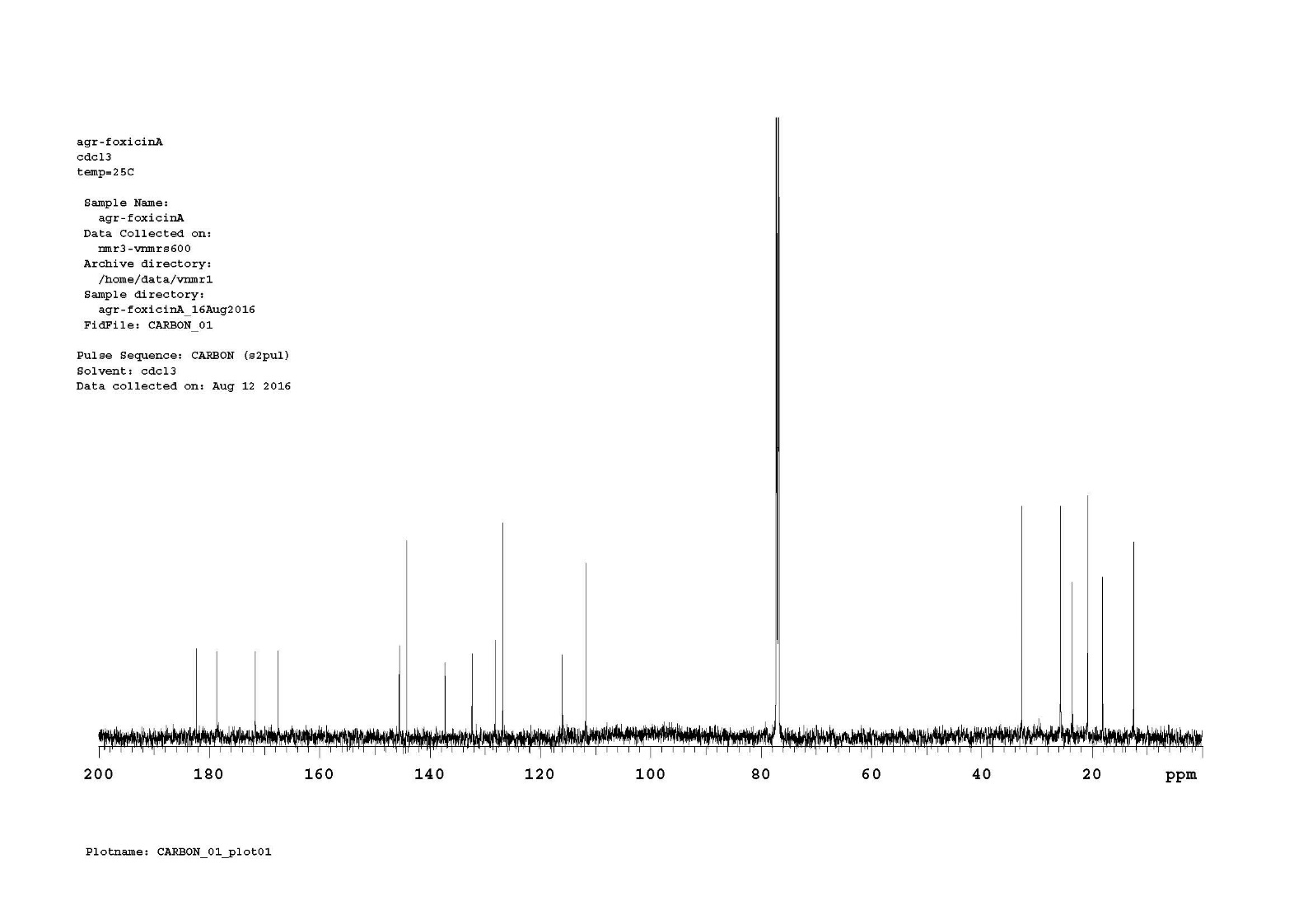


**Figure S3: ^13^C NMR of foxicin A (150MHz, CDCl_3_, 25 °C)**


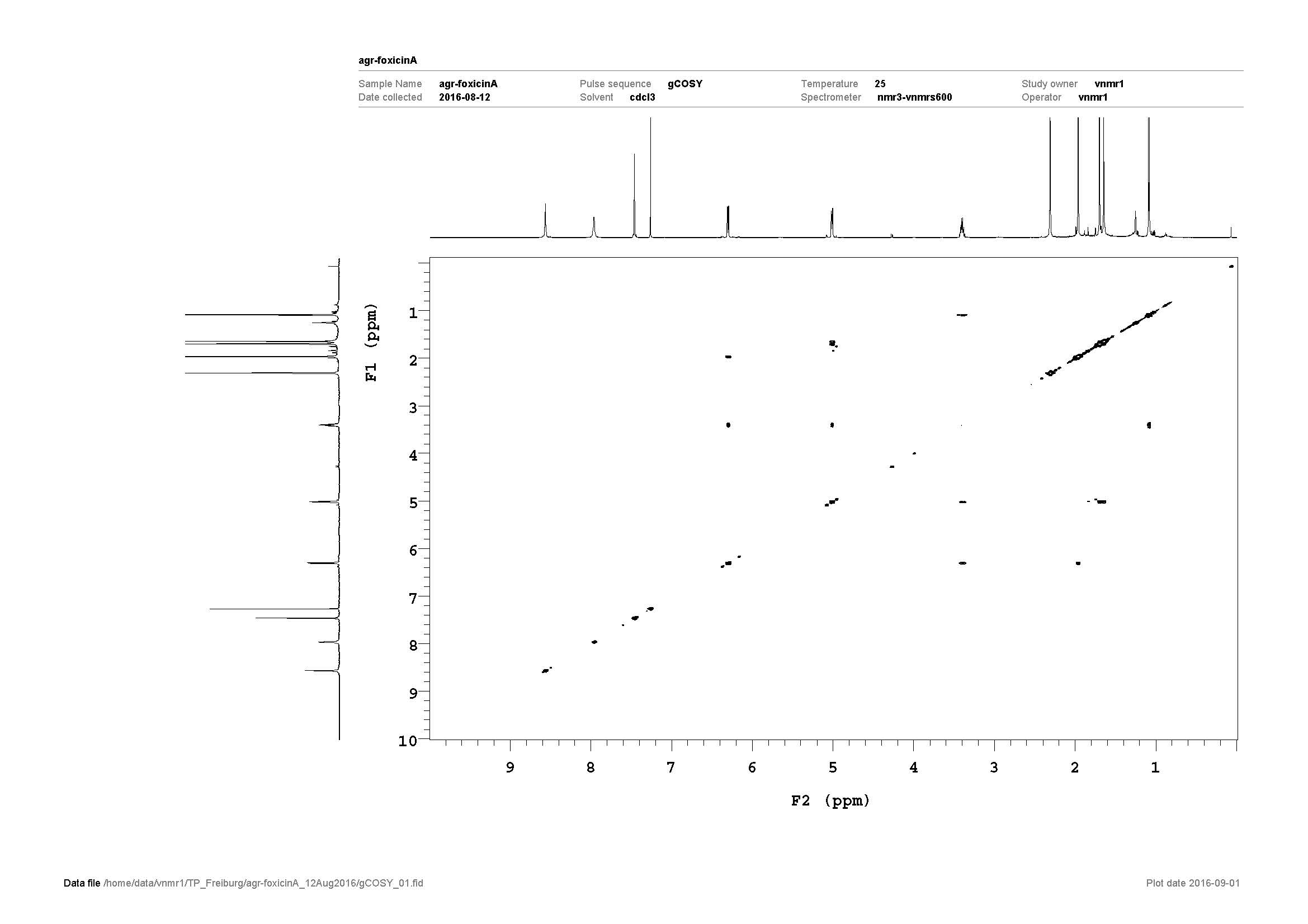


**Figure S4: COSY of foxicin A (600MHz, CDCl3, 25 °C)**


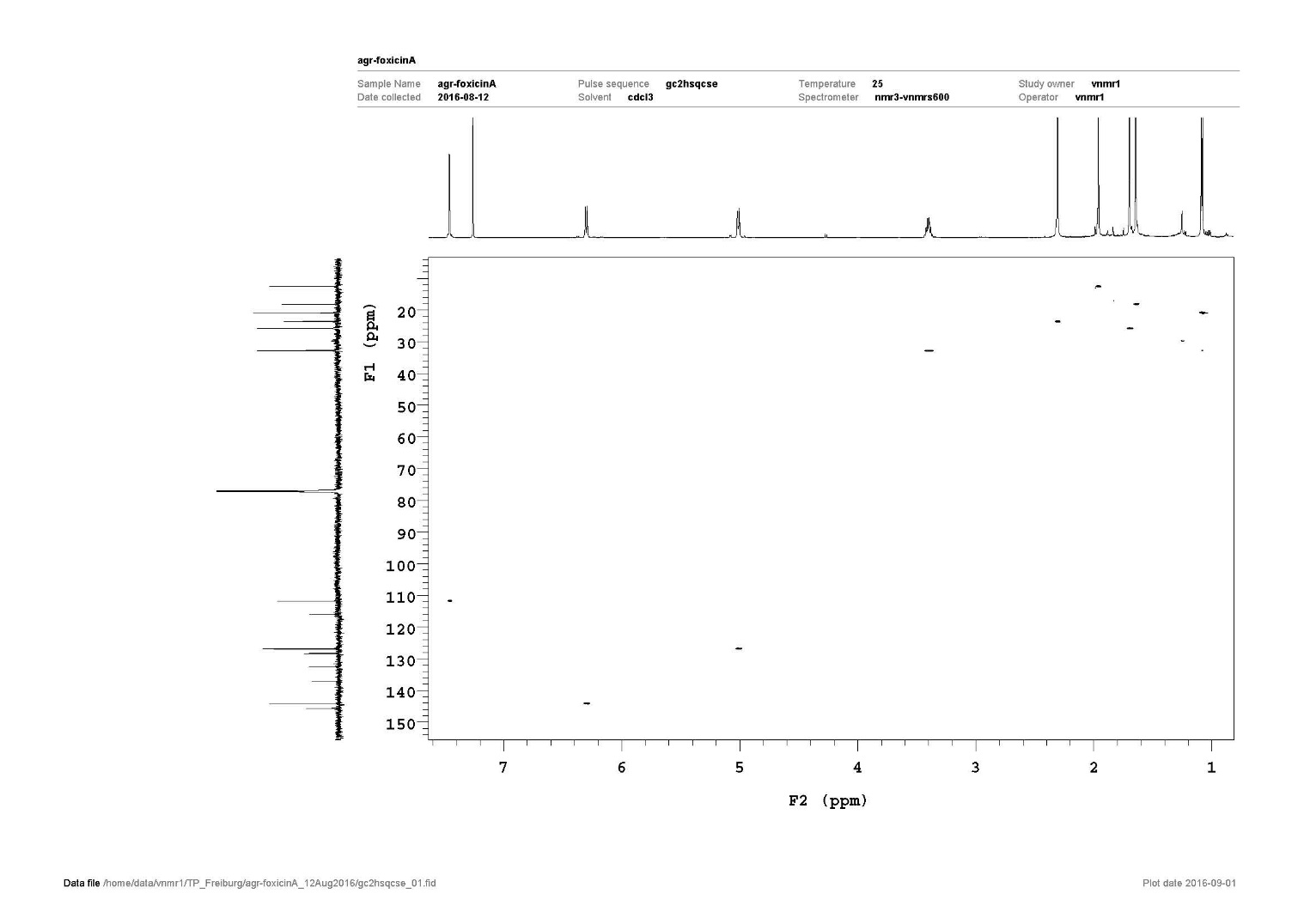


**Figure S5: ^1^H,^13^C-HSQC of foxicin A (600MHz, CDCl_3_, 25 °C)**

**Figure S6: ^1^H,^13^C-HMBC of foxicin A (600MHz, CDCl_3_, 25 °C)**


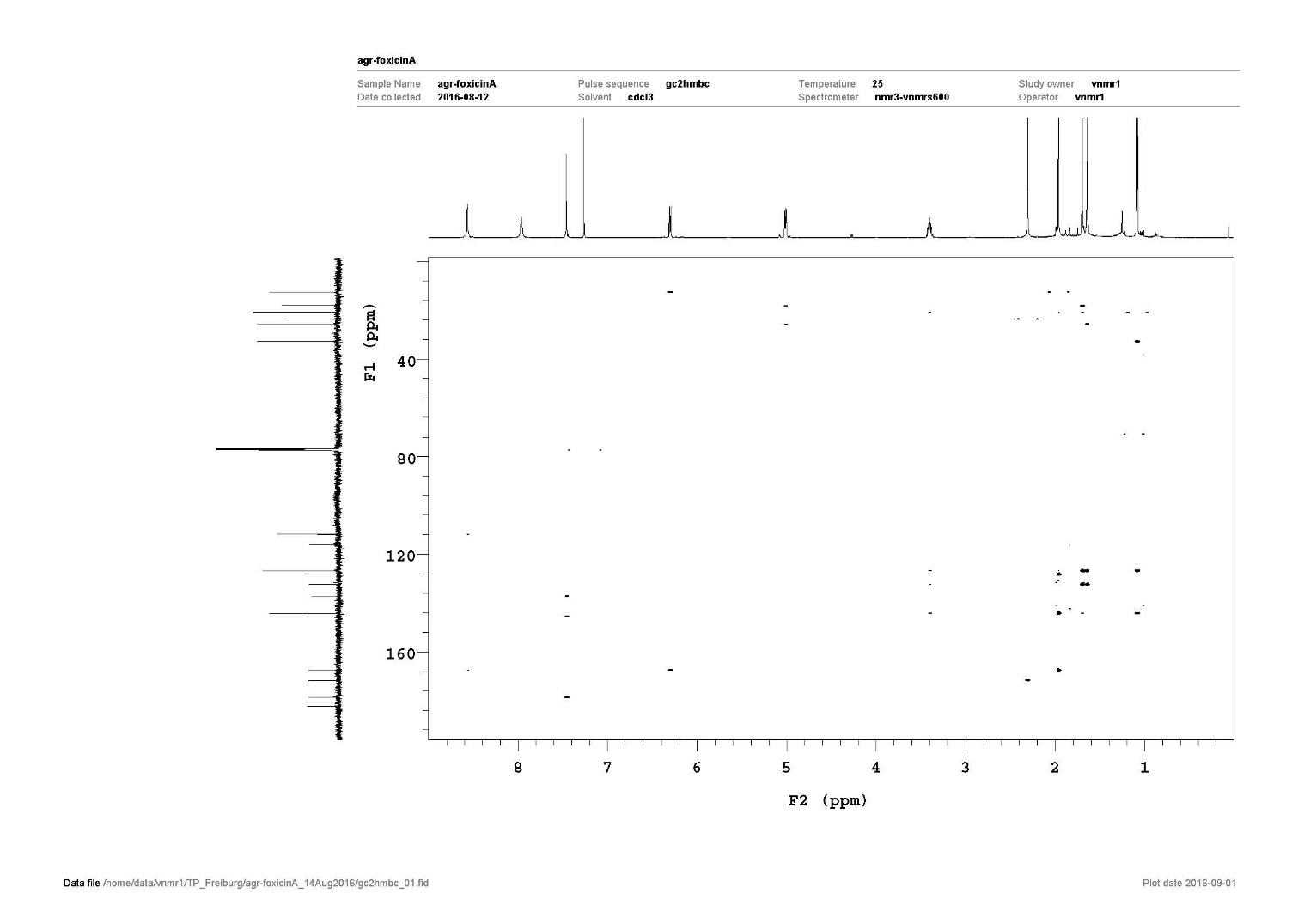


**Figure S7: ^1^H,^13^C-H2BC of foxicin A (600MHz, CDCl_3_, 25 °C)**


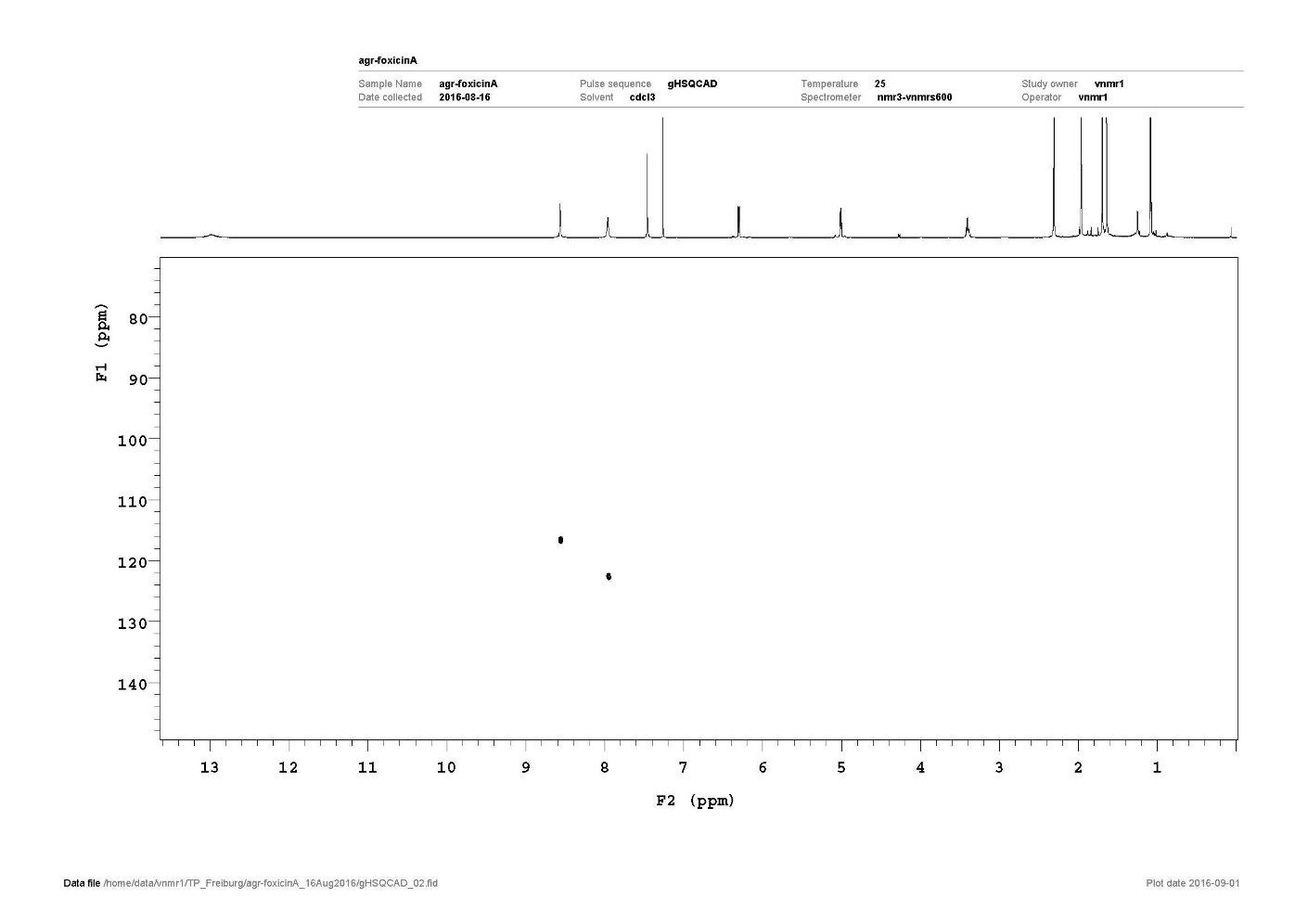


**Figure S8: ^1^H,^15^N-HSQC of foxicin A (600MHz, CDCl_3_, 25 °C)**


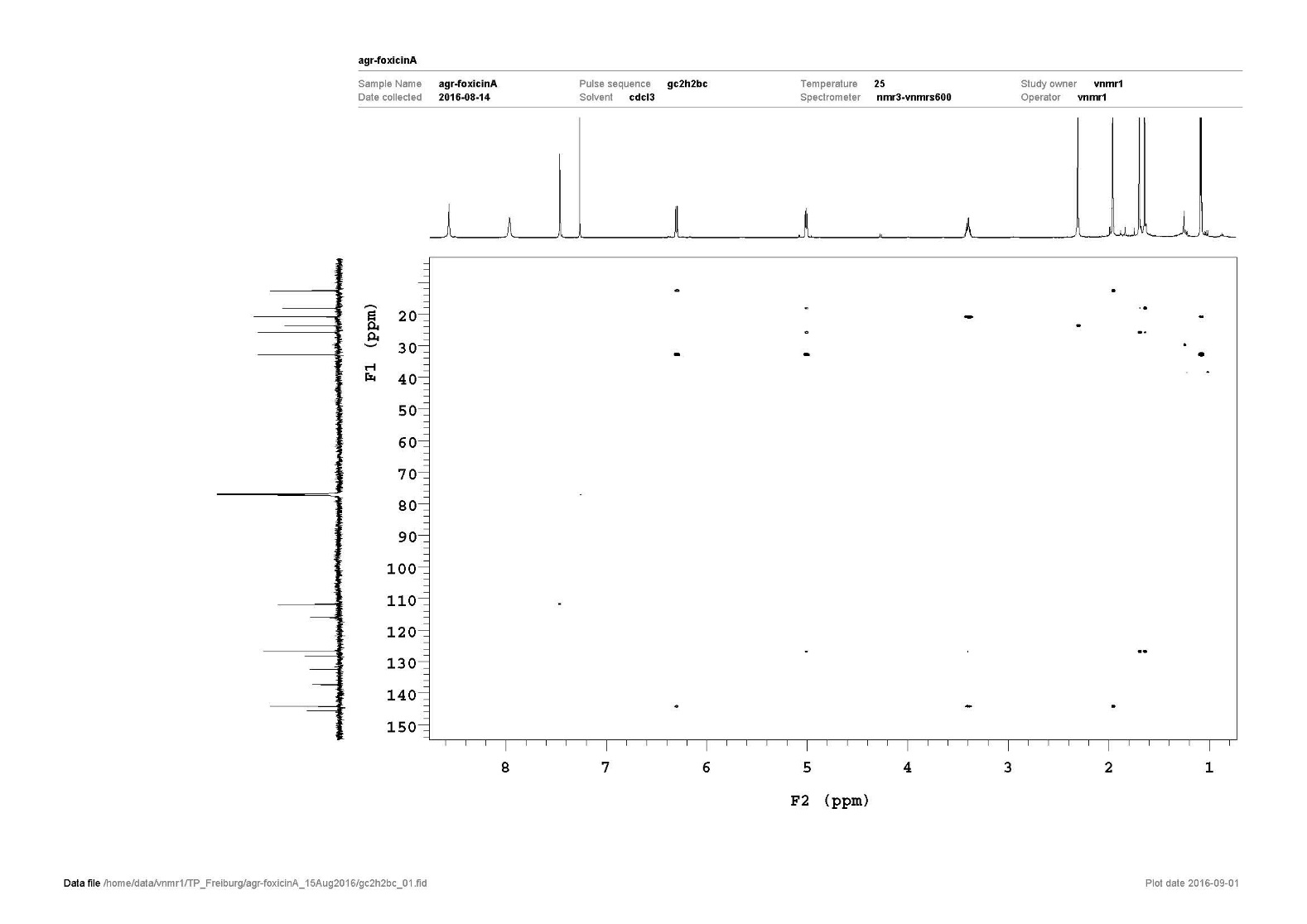


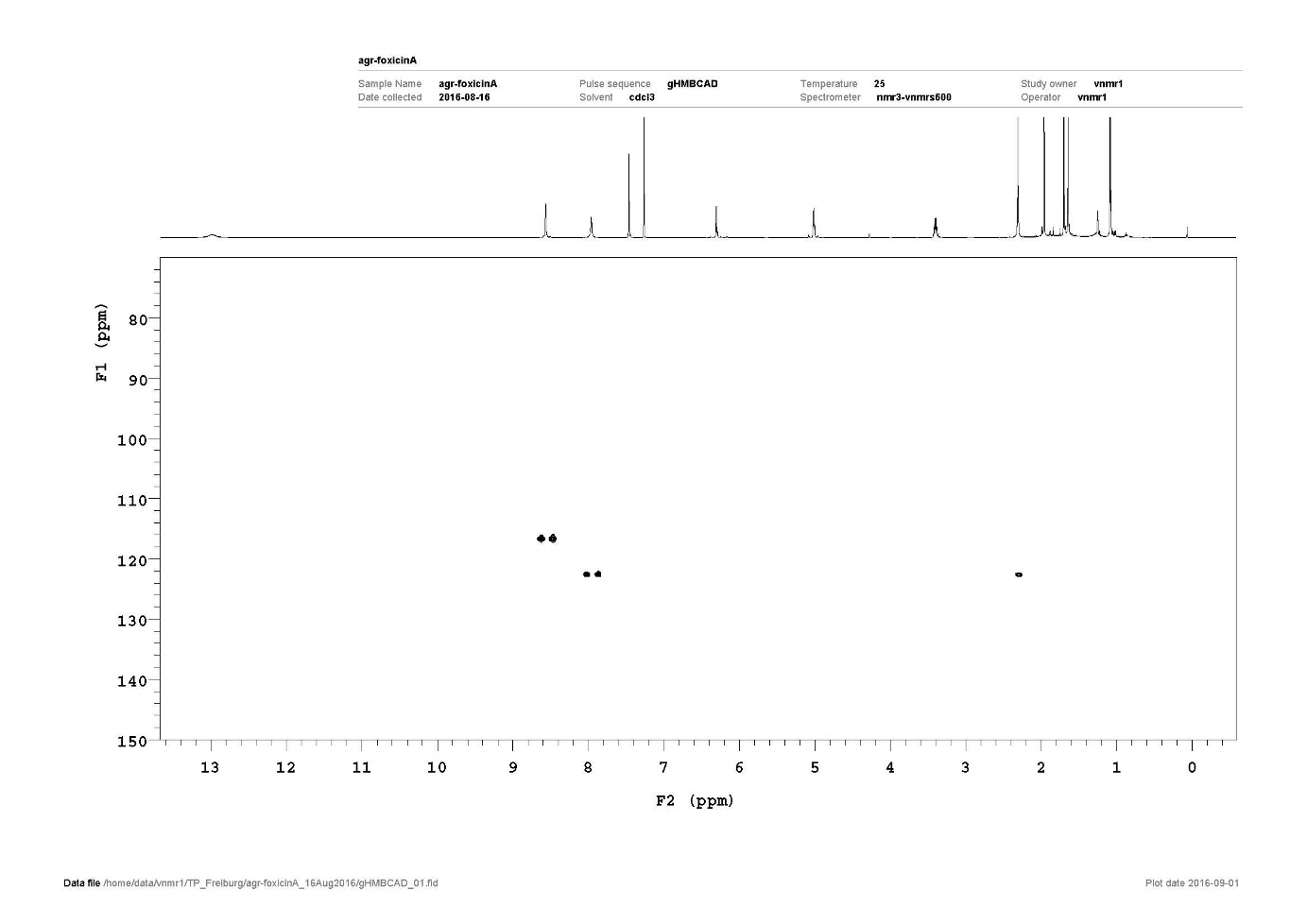
**Figure S9: ^1^H,^15^N-HMBC of foxicin A (600MHz, CDCl_3_, 25 °C)**

**Figure S10: ^1^H NMR of foxicin A (600MHz, DMSO-d_6_, 35 °C)**


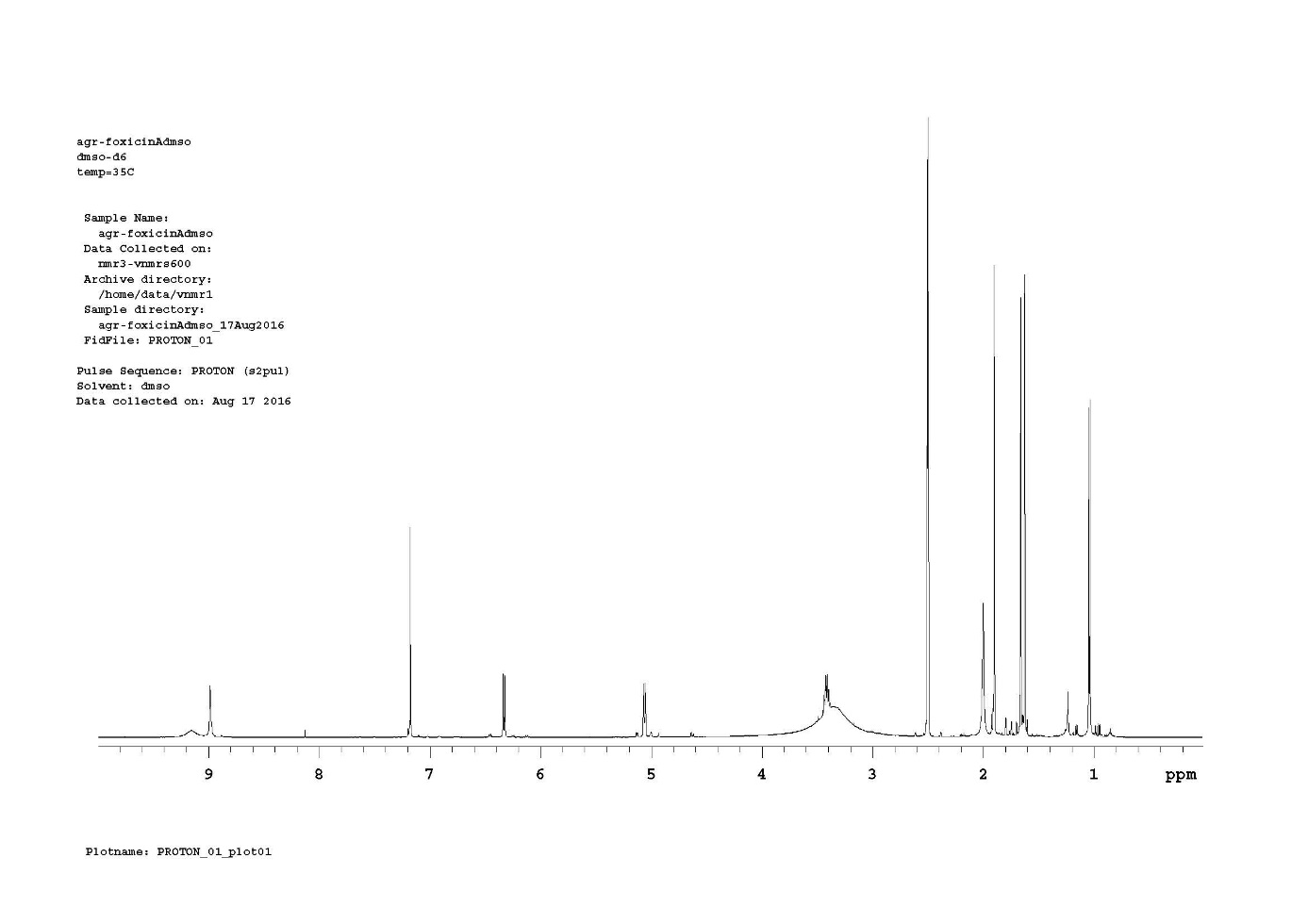


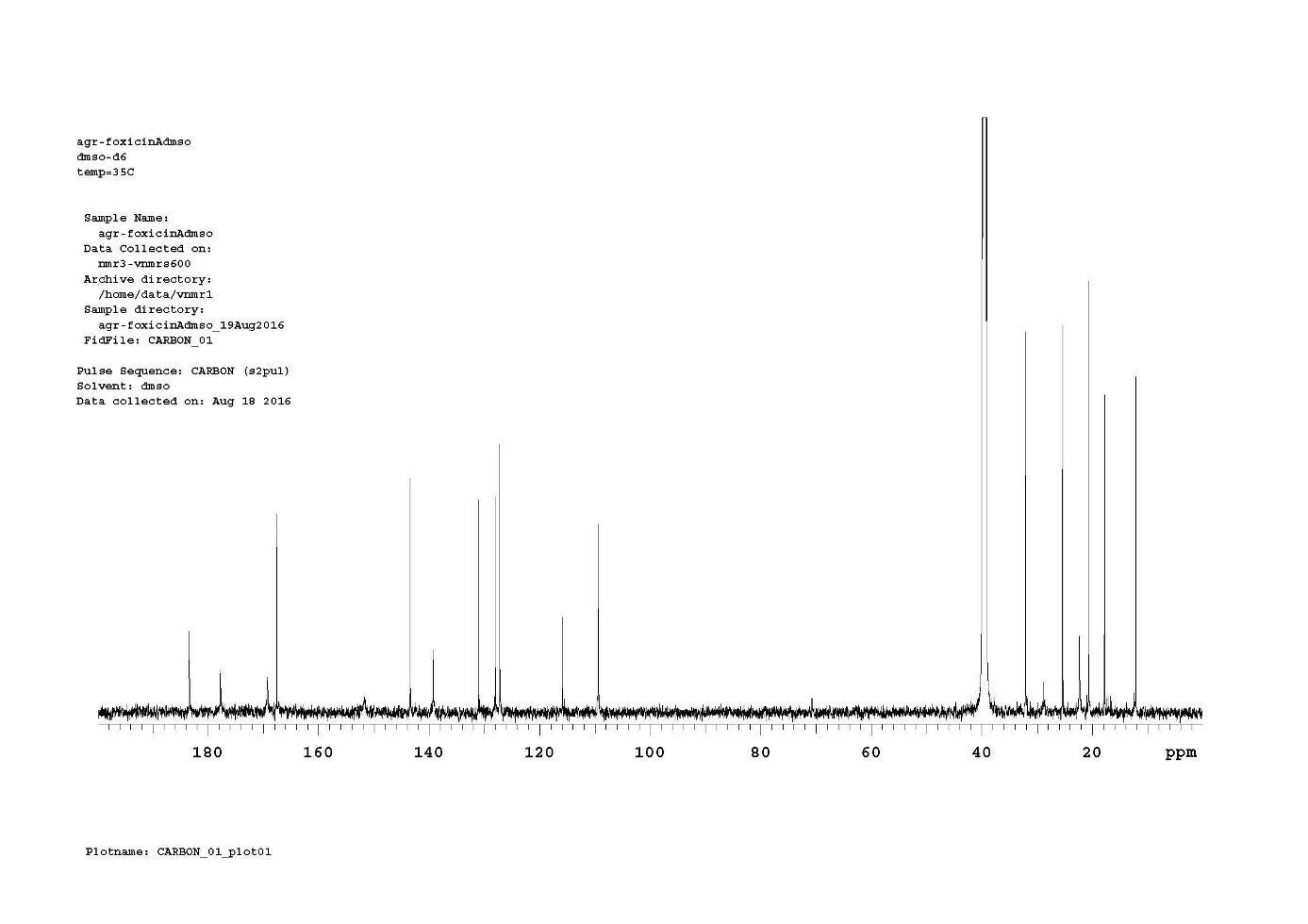


**Figure S11: ^13^C NMR of foxicin A (150MHz, DMSO-d_6_, 35 °C)**


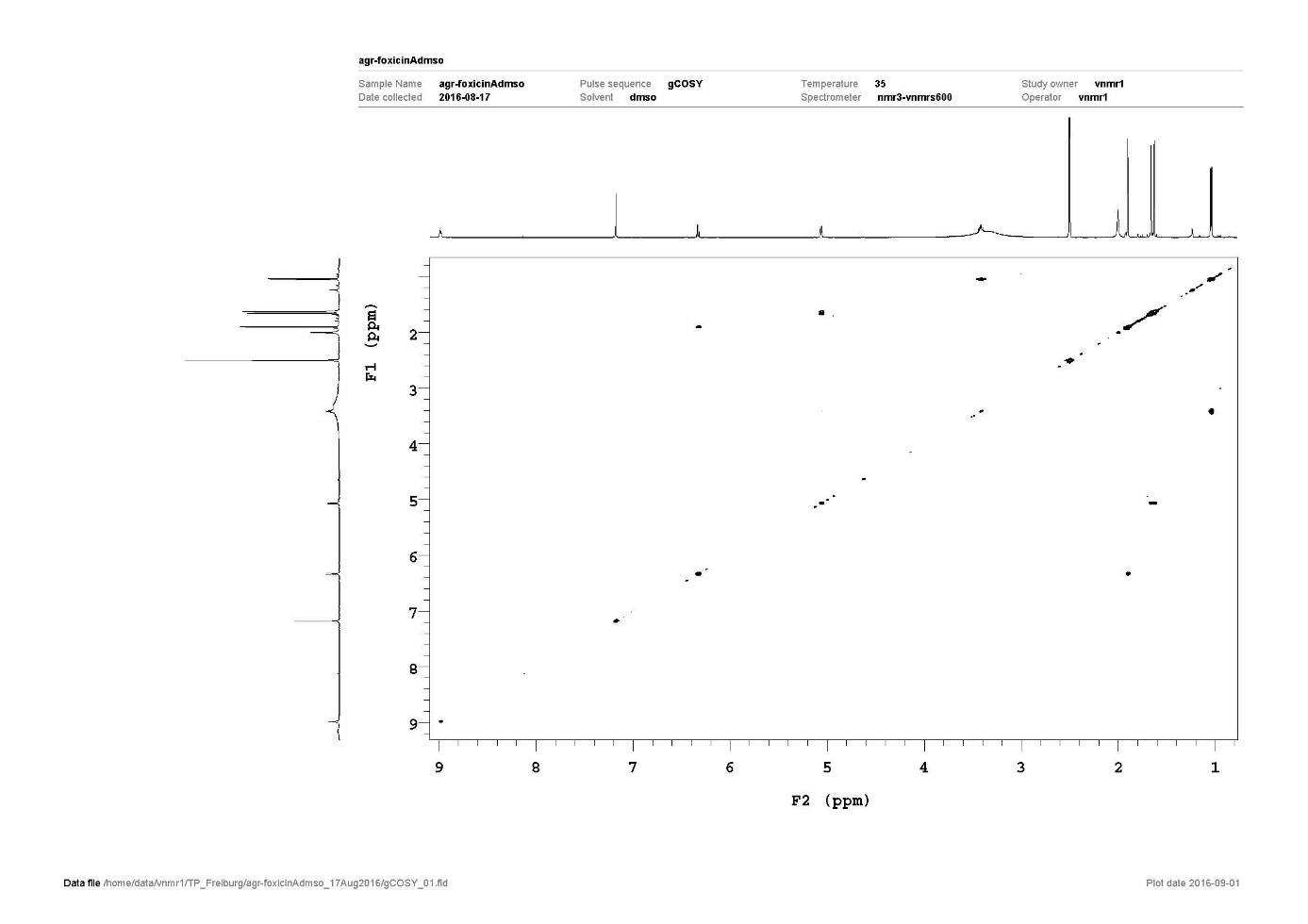


**Figure S12: COSY of foxicin A (600MHz, DMSO-d_6_, 35 °C)**


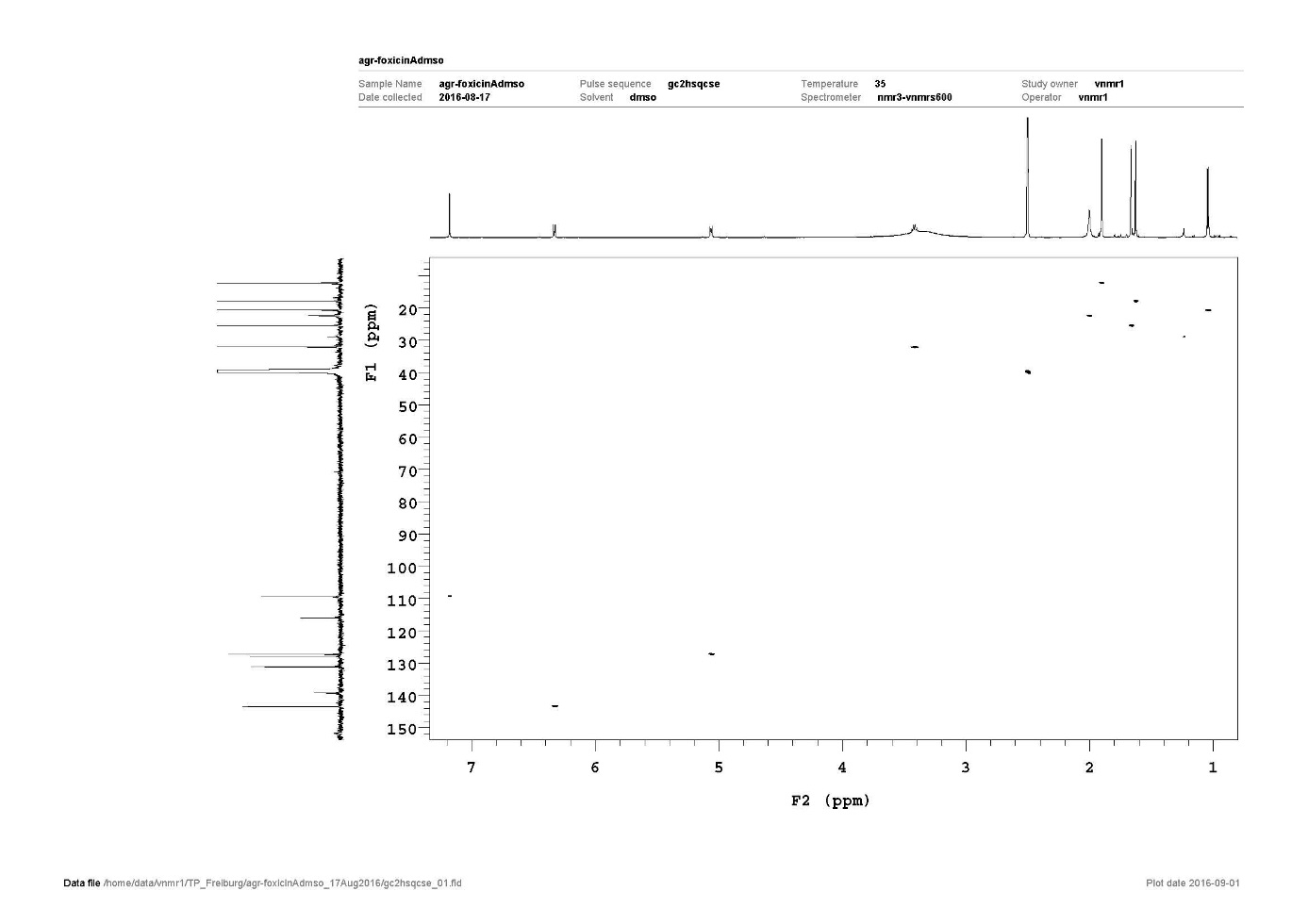


**Figure S13: ^1^H,^13^C-HSQC of foxicin A (600MHz, DMSO-d_6_, 35 °C)**


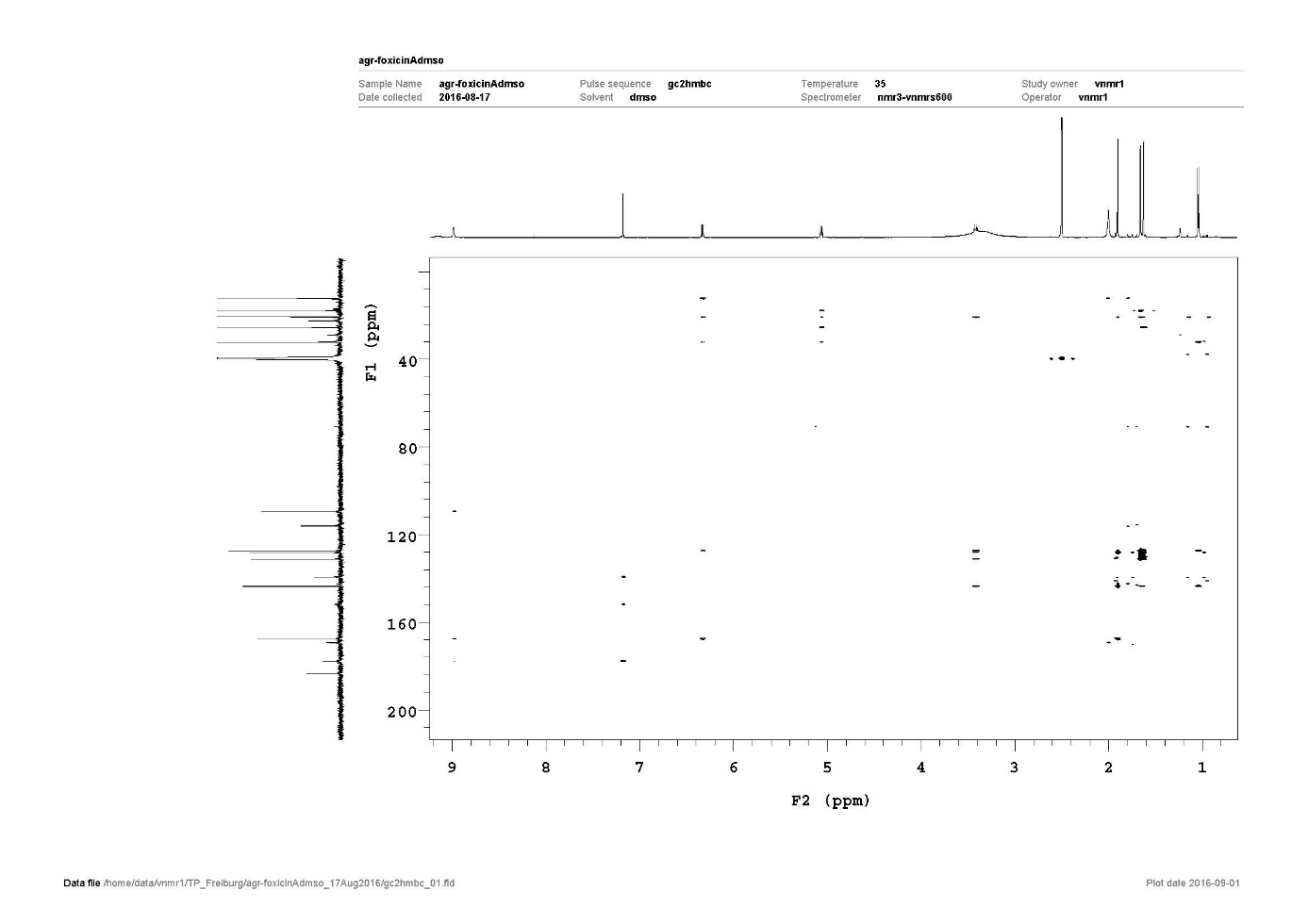


**Figure S14: ^1^H,^13^C-HMBC of foxicin A (600MHz, DMSO-d_6_, 35 °C)**

**Figure S15. Agar plate diffusion assay with H_2_O_2_**

**Figure S16: Cell viability of cancer cells and peripheral blood mononuclear cells (PBMC) from healthy human subjects after 24 h of incubation with foxicin A using the MTT assay.**

**3. Computer programs**

***anti*SMASH** (antibiotics & Secondary Metabolite Analysis Shell) were used to search for secondary metabolite gene clusters in the genome of *Streptomyces diastatochromogenes* Tü6028 and to identify the foxicin biosynthesis gene cluster (<http://antismash.secondarymetabolites.org/>) (Weber et al., 2015)

**BLAST** (Basic Local Alignment Search Tool) analysis was used to characterize foxicin biosynthesis gene cluster. BlastX was run to search the translated nucleotide query against a protein database (<http://blast.ncbi.nlm.nih.gov/>) (Altschul et al., 1990)

**Clustal Omega** analysis was used to align *foxBI*, *foxBII*, *foxBIII*, *foxEI* and *foxEII* homologous genes in other *Streptomyces* strains, to identify %identity and creating of the phylogenetic tree (<http://www.ebi.ac.uk/Tools/msa/clustalo/>) (Sievers et al., 2011)

**4. References**

Altschul, S. F., Gish, W., Miller, W., Myers, E. W., and Lipman, D. J. (1990). Basic local alignment search tool. *J. Mol. Biol.* 215, 403–10. doi:10.1016/S0022-2836(05)80360-2.

Burg, R. W., Miller, B. M., Baker, E. E., Birnbaum, J., Currie, S. A., Hartman, R., et al. (1979). Avermectins, new family of potent anthelmintic agents: producing organism and fermentation. *Antimicrob. Agents Chemother.* 15, 361–7. doi:10.1128/AAC.15.3.361.

Buzzetti, F., Eisenberg, F., Grant, H. N., Keller-Schierlein, W., Voser, W., and Zähner, H. (1968). Avilamycin. *Experientia* 24, 320–324.

Doroghazi, J. R., Albright, J. C., Goering, A. W., Ju, K.-S., Haines, R. R., Tchalukov, K. A., et al. (2014). A roadmap for natural product discovery based on large-scale genomics and metabolomics. *Nat. Chem. Biol.* 10, 963–8. doi:10.1038/nchembio.1659.

Florent, Y., Lunel, Y., and Mancy, D. (1967). Nouvelle substance antifonginique, sa preparation et les compositions qui la contiennet. *Fr. Pat.* Fr. 761114.

Gaisser, S., Trefzer, A., Stockert, S., Kirschning, A., and Bechthold, A. (1997). Cloning of an avilamycin biosynthetic gene cluster from *Streptomyces viridochromogenes* Tü57. *J. Bacteriol.* 179, 6271–8. doi: 10.1128/jb.179.20.6271-6278.1997.

Galmarini, O. L., and Deulofeu, V. (1961). Curamycin—I. *Tetrahedron* 15, 76–86. doi:10.1016/0040-4020(61)80010-0.

Gros, E. G., Deulofeu, V., Galmarini, O. L., and Frydman, B. (1968). Curamycin. II. Structure of the hydrolysis products “curacin” and “D-curamicose”. *Experientia* 24, 323–4. doi:10.1007/BF02140795.

Hamada, S. (1958). A Study of a New Antitumor Substance, Cellostatin. I. On the isolation and some properties of cellostatin. *Tohoku J. Exp. Med.* 67, 173–9. doi: 10.1620/tjem.67.173.

Hamada, S., and Sato, S. (1958). A Study of a New Antitumor Substance, Cellostatin. II. On antitumor effects of cellostatin. *Tohoku J. Exp. Med.* 67, 181–186. doi:10.1620/tjem.67.181.

Hendlin, D., Stapley, E. O., Jackson, M., Wallick, H., Miller, A. K., Wolf, F. J., et al. (1969). Phosphonomycin, a new antibiotic produced by strains of *Streptomyces*. *Science (80).* 166, 122–123. doi:10.1126/science.166.3901.122.

Iftime, D., Jasyk, M., Kulik, A., Imhoff, J. F., Stegmann, E., Wohlleben, W., et al. (2015). Streptocollin, a Type IV lanthipeptide produced by *Streptomyces collinus* Tü365. *Chembiochem* 16, 2615–23. doi:10.1002/cbic.201500377.

Iftime, D., Kulik, A., Härtner, T., Rohrer, S., Niedermeyer, T. H. J., Stegmann, E., et al. (2016). Identification and activation of novel biosynthetic gene clusters by genome mining in the kirromycin producer *Streptomyces collinus* Tü365. *J. Ind. Microbiol. Biotechnol.* 43, 277–91. doi:10.1007/s10295-015-1685-7.

Ju, K.-S., Gao, J., Doroghazi, J. R., Wang, K.-K. A., Thibodeaux, C. J., Li, S., et al. (2015). Discovery of phosphonic acid natural products by mining the genomes of 10,000 actinomycetes. *Proc. Natl. Acad. Sci. U. S. A.* 112, 12175–80. doi:10.1073/pnas.1500873112.

Oshima, K., Hattori, M., Shimizu, H., Fukuda, K., Nemoto, M., Inagaki, K., et al. (2015). Draft genome sequence of *Streptomyces incarnatus* NRRL8089, which produces the nucleoside antibiotic sinefungin. *Genome Announc.* 3. doi:10.1128/genomeA.00715-15.

Ser, H.-L., Palanisamy, U. D., Yin, W.-F., Abd Malek, S. N., Chan, K.-G., Goh, B.-H., et al. (2015). Presence of antioxidative agent, pyrrolo[1,2-a]pyrazine-1,4-dione, hexahydro- in newly isolated *Streptomyces mangrovisoli* sp. nov. *Front. Microbiol.* 6, 854. doi:10.3389/fmicb.2015.00854.

Sievers, F., Wilm, A., Dineen, D., Gibson, T. J., Karplus, K., Li, W., et al. (2011). Fast, scalable generation of high-quality protein multiple sequence alignments using Clustal Omega. *Mol. Syst. Biol.* 7, 539. doi:10.1038/msb.2011.75.

Wang, X.-J., Wang, J.-D., Xiang, W.-S., and Zhang, J. (2009). Three new milbemycin derivatives from *Streptomyces bingchenggensis*. *J. Asian Nat. Prod. Res.* 11, 597–603. doi:10.1080/10286020902819897.

Weber, T., Blin, K., Duddela, S., Krug, D., Kim, H. U., Bruccoleri, R., et al. (2015). antiSMASH 3.0 - a comprehensive resource for the genome mining of biosynthetic gene clusters. *Nucleic Acids Res.* 43, W237-43. doi:10.1093/nar/gkv437.

Weber, T., Laiple, K. J., Pross, E. K., Textor, A., Grond, S., Welzel, K., et al. (2008). Molecular analysis of the kirromycin biosynthetic gene cluster revealed beta-alanine as precursor of the pyridone moiety. *Chem. Biol.* 15, 175–88. doi:10.1016/j.chembiol.2007.12.009.

White, R. F., and Demain, L. A. (1976). (-)(Cis-1,2-epoxy propyl)phosphonic acid amides. US 3943153 A.

Wolf, H., and Zähner, H. (1972). Metabolic products of microorganisms. 99. Kirromycin. *Arch. für Mikrobiol.* 83, 147–54.

Xiang, W.-S., Wang, J.-D., Wang, X.-J., and Zhang, J. (2009). Bingchamides A and B, two novel cyclic pentapeptides from the *Streptomyces bingchenggensis*: fermentation, isolation, structure elucidation and biological properties. *J. Antibiot. (Tokyo).* 62, 501–5. doi:10.1038/ja.2009.60.

Zhang, J., An, J., Wang, J.-J., Yan, Y.-J., He, H.-R., Wang, X.-J., et al. (2013). Genetic engineering of *Streptomyces bingchenggensis* to produce milbemycins A3/A4 as main components and eliminate the biosynthesis of nanchangmycin. *Appl. Microbiol. Biotechnol.* 97, 10091–101. doi:10.1007/s00253-013-5255-5.

Zhu, H., Guo, J., Yao, Q., Yang, S., Deng, M., and Li, T. (2011). *Streptomyces caeruleatus* sp. nov., with dark blue diffusible pigment. *Int. J. Syst. Evol. Microbiol.* 61, 507–11. doi:10.1099/ijs.0.017392-0.
